# Prevalence and antimicrobial resistance profiles of *Vibrio* spp. and *Enterococcus* spp. in retail shrimp in Northern California

**DOI:** 10.3389/fmicb.2023.1192769

**Published:** 2023-06-28

**Authors:** Brady Hirshfeld, Kurtis Lavelle, Katie Yen Lee, Edward Robert Atwill, David Kiang, Bakytzhan Bolkenov, Megan Gaa, Zhirong Li, Alice Yu, Xunde Li, Xiang Yang

**Affiliations:** ^1^Department of Animal Science, University of California, Davis, Davis, CA, United States; ^2^Western Institute for Food Safety and Security, University of California, Davis, Davis, CA, United States; ^3^Department of Population Health and Reproduction, School of Veterinary Medicine, University of California, Davis, Davis, CA, United States; ^4^California Department of Public Health, Richmond, CA, United States

**Keywords:** *Vibrio* spp., *Enterococcus* spp., foodborne, shrimp, antimicrobial resistance, whole genome sequencing, resistance genes

## Abstract

Shrimp is one of the most consumed seafood products globally. Antimicrobial drugs play an integral role in disease mitigation in aquaculture settings, but their prevalent use raises public health concerns on the emergence and spread of antimicrobial resistant microorganisms. *Vibrio* spp., as the most common causative agents of seafood-borne infections in humans, and *Enterococcus* spp., as an indicator organism, are focal bacteria of interest for the monitoring of antimicrobial resistance (AMR) in seafood. In this study, 400 samples of retail shrimp were collected from randomly selected grocery stores in the Greater Sacramento, California, area between September 2019 and June 2020. The prevalence of *Vibrio* spp. and *Enterococcus* spp. was 60.25% (241/400) and 89.75% (359/400), respectively. Subsamples of *Vibrio* (*n* = 110) and *Enterococcus* (*n* = 110) isolates were subjected to antimicrobial susceptibility testing (AST). *Vibrio* isolates had high phenotypic resistance to ampicillin (52/110, 47.27%) and cefoxitin (39/110, 35.45%). *Enterococcus* were most frequently resistant to lincomycin (106/110, 96.36%), quinupristin-dalfopristin (96/110, 87.27%), ciprofloxacin (93/110, 84.55%), linezolid (86/110, 78.18%), and erythromycin (58/110, 52.73%). For both *Vibrio* and *Enterococcus*, no significant associations were observed between multidrug resistance (MDR, resistance to ≥3 drug classes) in isolates from farm raised and wild caught shrimp (*p* > 0.05) and in isolates of domestic and imported origin (*p* > 0.05). Whole genome sequencing (WGS) of a subset of *Vibrio* isolates (*n* = 42) speciated isolates as primarily *V. metschnikovii* (24/42; 57.14%) and *V. parahaemolyticus* (12/42; 28.57%), and detected 27 unique antimicrobial resistance genes (ARGs) across these isolates, most commonly *qnrVC6* (19.05%, 8/42), dfrA31 (11.90%, 5/42), dfrA6 (9.5%, 4/42), *qnrVC1* (9.5%, 4/42). Additionally, WGS predicted phenotypic resistance in *Vibrio* isolates with an overall sensitivity of 11.54% and specificity of 96.05%. This study provides insights on the prevalence and distribution of AMR in *Vibrio* spp. and *Enterococcus* spp. from retail shrimp in California which are important for food safety and public health and exemplifies the value of surveillance in monitoring the spread of AMR and its genetic determinants.

## 1. Introduction

Shrimp is the most popular seafood in the United States (U.S.) and is vaunted by the United States Department of Agriculture (USDA) as a healthy protein due to its nutrient density, lower unhealthy fat content relative to red meat and poultry, and lower levels of methylmercury compared to many other seafoods [U.S. Department of Agriculture and U.S. Department of Health and Human Services (USDA and USDHHS), 2020; National Fisheries Institute Media, 2022]. Annual per-capita consumption of shrimp in the U.S. approached five pounds in 2020, despite nearly 90% of Americans eating less than the recommended quantity of shrimp [U.S. Department of Agriculture and U.S. Department of Health and Human Services (USDA and USDHHS), 2020; National Marine Fisheries Service, 2022; Thomsen et al., 2022]. Still, the U.S. is one of the largest importers in the global shrimp industry which was valued at USD 24.7 billion in 2022 and is still growing at an accelerating rate [National Fisheries Institute Media, 2022; Food and Agriculture organization of the United Nations (FAO), 2022a].

Shrimp aquaculture, which already outproduces wild shrimp fisheries three times over, is the fastest growing animal food sector in the world [Kumar et al., 2016; Golder et al., 2022; Food and Agriculture Organization of the United Nations (FAO), 2022b]. The majority of shrimp production occurs in countries in Asia (primarily China, Thailand, Indonesia, and India) and South and Central America (especially Ecuador) [Food and Agriculture Organization of the United Nations (FAO), 2022b]. Farm raised shrimp are highly susceptible to infectious disease due to high stocking densities and their decreased capacity for adaptive immunity relative to vertebrates (Ali et al., 2018; Thornber et al., 2020; Lanz-Mendoza and Contreras-Garduño, 2022). Outbreaks can endanger entire harvests without quick and aggressive treatment. Traditional vaccination, which has gained traction in finfish aquaculture, is not an option for shrimp, while new DNA-based vaccination methods have limited and poorly understood efficacy (Gudding and Van Muiswinkel, 2013; Chang et al., 2018). Consequentially, bacterial infectious diseases in farmed shrimp are almost always treated with antimicrobial agents. Since the scale of the industry and the risks posed by those diseases are so great, substantial volumes of antimicrobial drugs are used in shrimp production. From previous reports, 2.7% of all global antimicrobial usage of any type is attributable to shrimp aquaculture (Schar et al., 2020; Thornber et al., 2020).

The most common form of antimicrobial use in shrimp farming is feed-mediated metaphylaxis after the detection of an infection (Thornber et al., 2020). Prophylactic use of antimicrobials in shrimp aquaculture was once commonplace and is an ongoing practice, especially in hatchery settings, but has generally declined through the 21st century (Holmström et al., 2003; Zhang et al., 2011; Smith, 2012; Thornber et al., 2020). While antimicrobials are the first line treatment against pathogens in food production and clinical contexts, their use and misuse can increase selective pressures that lead to antimicrobial resistance (AMR) in bacteria [Centers for Disease Control and Prevention (CDC), 2019a]. Shrimp-associated bacteria are thus at high risk of developing AMR, which reduces the ability to treat infections that compromise animal welfare, human health, and industry. Moreover, since antimicrobial resistance genes (ARGs) can spread via horizontal gene transfer, even non-pathogenic bacteria – or those that are pathogenic for different hosts – that develop resistance can spread resistance to bacteria of greater public health concern (Lulijwa et al., 2020). Monitoring the prevalence and patterns of AMR in shrimp is therefore critical to evaluate food safety and public health risks.

Recently, a study conducted through the National Antimicrobial Resistance Monitoring System (NARMS) detected *Vibrio* spp. and *Enterococcus* spp. as the most prevalent Gram-negative and Gram-positive bacteria in retail seafood samples, respectively, and highlighted them as good candidates for tracking AMR (Tate et al., 2022). *Vibrio* spp. are cosmopolitan and are normal flora in the coastal and estuarine habitats of wild shrimp, but certain species are also the most common seafood-borne pathogens in humans (Costa et al., 2015a; Stratev et al., 2023) and the main pathogens for shrimp (El-Far et al., 2015; Amatul-Samahah et al., 2020). *Enterococcus* spp. are primarily commensal bacteria in the environment and animal gut microbiomes, but are often employed as indicator organisms for the monitoring of antimicrobial resistance since they can readily acquire ARGs conferring resistance to a large diversity of antimicrobials and transfer them to other bacteria, including pathogens (Byappanahalli et al., 2012; Chajęcka-Wierzchowska et al., 2016; Çardak et al., 2022). The objective of this study was to evaluate the prevalence and antimicrobial resistance profiles of *Vibrio* spp. and *Enterococcus* spp. in retail shrimp from grocery stores in the greater Sacramento area in California.

## 2. Materials and methods

### 2.1. Sample collection

A total of 400 shrimp samples, either prepackaged or in half-pound packages from bulk seafood counters, were collected from grocery stores in the Greater Sacramento area in California over four seasonal periods between September 2019 and June 2020. A list of 100 grocery stores was randomly selected among those located in Sacramento zip codes according to Google Maps. During each sampling event, stores were randomly selected from this pool. Samples were randomly selected from chilled retail displays in the manner they were presented to consumers. Along with the samples, metadata including production type (farm raised or wild caught), country of origin, species and size of shrimp, store handling method, sold forms (fresh, frozen, or previously frozen), and time of collection were collected during sampling. Samples were kept on ice during transport, refrigerated upon receipt at the laboratory, and processed within 72 h of collection.

### 2.2. Sample processing, bacterial isolation, and confirmation

Samples were processed using the NARMS seafood pilot laboratory protocol (FDA, 2021). Briefly, two aliquots of 25 g from each shrimp sample were placed into two sterile stomacher bags, one containing 225 mL of alkaline peptone water (APW) and another with buffered peptone water (BPW). Samples were homogenized in a Neutec Masticator Paddle Blender (Neutec Group, Inc., Farmingdale, NY, United States) for 2 min at 230 RPM and incubated at 35°C for 24 ± 2 h. Subsequently, overnight APW and BPW enrichments were streaked onto thiosulfate-citrate-bile salts-sucrose (TCBS) (BD Difco, Detroit, MI, United States) and Enterococcosel (BD BBL, Franklin Lakes, NJ, United States) agars, respectively, and incubated at 35°C for 18–24 h for identification of *Vibrio* spp. and *Enterococcus* spp., respectively. One colony with positive colony morphology (yellow or green to blue-green colonies being characteristic of *Vibrio*, and beige colonies with strong black halos being characteristic of *Enterococcus*) was selected from each plate and streaked to purity on blood agar plates. Presumptive positives for Vibrio were confirmed to genus level by PCR using the forward primer: 5’-GGC GTA AAG CGC ATG CAG GT-3′; and the reverse primer: 5′-GAA ATT CTA CCC CCC TCT ACA G-3′, as previously described in Thompson et al. (2004). *Enterococcus* were confirmed with Gram-staining for identification of Gram-positive cocci and biochemical tests (catalase negative and PYR positive) using BD BBL DrySlide™ PYR kits and following methods previously described by Aryal (2016).

### 2.3. Antimicrobial susceptibility testing

Antimicrobial susceptibility testing (AST) was conducted on a subset of isolates comprised of 110 *Vibrio* (110/241) and 110 *Enterococcus* (110/359) isolates using the broth microdilution method with the NARMS Gram-negative (CMV3AGNF) and Gram-positive (CMV3AGPF) panels, respectively. Isolates were streaked onto selective agar plates (TCBS and Enterococcosel agar for *Vibrio* and *Enterococcus*, respectively) and incubated at 35°C for 18–24 h. A colony with typical morphology was then restreaked onto blood agar (Thermo Fisher Scientific, Waltham, MA, United States) and incubated at 35°C for 20–24 h. Pure colonies on fresh overnight blood agar plates were suspended in sterile demineralized water to an optical density (OD) between 0.08 and 0.10 as measured by a spectrophotometer (BioMate 3; ThermoSpectronic, Rochester, NY) at 625 nm. Aliquots of the suspension (20 uL for *Vibrio* and 10 uL for *Enterococcus*) were then transferred to 11 mL of cation-adjusted Mueller-Hinton broth (CAMHB; BD Difco, Detroit, MI, United States), and the mixture was vortexed for 5–10 s. Subsequently, 50 uL of the CAMHB mixture was transferred to each well of the AST plate. Additionally, a loopful (10 uL) of the CAMHB suspension was streaked onto a blood agar plate for quality control. AST plates and blood agar plates were then incubated at 35°C for 18–24 h. The minimum inhibitory concentration (MIC) was recorded as the lowest concentration of each drug with fully inhibited growth in the wells and per guidelines from Clinical and Laboratory Standards Institute (CLSI) methods [Clinical and Laboratory Standards Institute (CLSI), 2018].

Resulting MIC values were interpreted as susceptible, resistant, or intermediate based on CDC breakpoints for non-cholera *Vibrio* [Centers for Disease Control and Prevention (CDC), 2019a] and FDA NARMS breakpoints for *Enterococcus* [U.S. Food and Drug Administration (FDA), 2019], both of which are based on CLSI breakpoints [Clinical and Laboratory Standards Institute (CLSI), 2017]. Six of the fourteen drugs in the Gram-negative panel for *Vibrio* isolates (ceftiofur, ceftriaxone, chloramphenicol, nalidixic acid, streptomycin, and sulfisoxazole) have no CLSI or NARMS breakpoints, and were omitted from analysis. The composition of each drug panel and interpretive breakpoints for each drug are listed in Supplementary Table 1. Intermediate results were counted as resistant in the analysis. Multidrug resistance (MDR) was defined as resistance to three or more classes of antimicrobial drugs (Tate et al., 2022).

### 2.4. Whole genome sequencing (WGS) and identification of antimicrobial resistance genes (ARGs)

Whole genome sequencing was conducted on a subset of *Vibrio* isolates that exhibited phenotypic resistance (*n* = 42) at the Food and Drug Laboratory Branch of the California Department of Public Health. The isolates were streaked onto Trypticase Soy Agar with 0.6% Yeast Extract (TSA-YE) and 3% saline for recovery as well as CHROM *Vibrio* plates for confirmation. A single colony was restreaked on TSA-YE with 3% saline and incubated at 35°C for 18–24 h. Genomic DNA was extracted from bacteria using the DNeasy Blood and Tissue Kit (Qiagen) and quantified using a Qubit fluorometer (Thermo Fisher Scientific). DNA libraries were prepared with Illumina DNA Prep kits (Illumina Inc., San Diego, California). Whole genome sequencing was performed on the Illumina MiSeq DNA sequencing system using the MiSeq reagent kit version 2 (2 × 250-bp paired-end reads) per CDC PulseNet guidelines (PulseNet, n.d.). After the successful completion of the sequencing runs, the FASTQ files along with the corresponding metadata were submitted to the PulseNet for data analysis and uploaded to NCBI. Identification of antimicrobial resistance genes was done with raw reads using the ResFinder database (version 4.1, Center for Genetic Epidemiology, Kongens Lyngby, Denmark) with genes determined as present if sequences met thresholds of 90% identity and 60% minimum length (Zankari et al., 2017; Clausen et al., 2018; Bortolaia et al., 2020).

### 2.5. Statistical analysis

Descriptive statistics for the prevalence of *Vibrio* and *Enterococcus* in shrimp samples, the distribution of resistant patterns among isolates, sample characteristics, and the prevalence of resistance genes were conducted in Microsoft Excel (version 2,207, Redmond, WA, U.S.). Percent of isolates resistant to an antimicrobial agent was determined by dividing the number of isolates with a MIC value classified as resistant based on the appropriate CDC or FDA breakpoint criteria by the total number of isolates.

Prevalence and metadata analyses were performed using R version 4.1.2 (Vienna, Austria). Fisher’s exact test with adjusted *p*-values was used to evaluate the associations between these demographic factors and multidrug resistance. An *α* value of 0.05 was used to determine statistical significance for all analyses. Figures were created in R using packages ggplot2, ggtext, and heatmap.3.

Concordance between phenotypic resistance from AST and genotypic resistance from ARGs identified through WGS were evaluated for non-cholera *Vibrio* isolates (*n* = 40) for gentamicin, amoxicillin/clavulanic acid 2:1 ratio, cefoxitin, trimethoprim/sulfamethoxazole, azithromycin, ampicillin, and tetracycline as previously described (Lee et al., 2022). Phenotype and genotype were considered concordant when an isolate with phenotypic resistance to a drug in the MIC panel also had ARGs associated with the corresponding drug (true positive, TP), or when an isolate with phenotypic susceptibility to a drug also did not contain any corresponding ARGs (true negative, TN). TP and TN results indicated that *in-silico* predictions of resistance based on WGS were concordant with phenotypic observations. False negatives (FN) were defined as isolates that exhibited phenotypic resistance but did not harbor any ARGs known to confer resistance to the corresponding drug, and false positives (FP) were defined as isolates that exhibited phenotypic susceptibility to a drug but contained ARGs associated with that drug. FP and FN results indicated discordance between *in-silico* predictions and observed phenotypic resistance. Sensitivity was calculated as TP/(TP + FN) and specificity was calculated as TN/(TN + FP). Two *Vibrio* isolates that were speciated as *V. cholerae* were omitted from concordance analysis because phenotypic resistance was determined based on MIC breakpoints defined specifically for non-cholera *Vibrio*.

## 3. Results

### 3.1. Prevalence of *Vibrio* spp. and *Enterococcus* spp. in retail shrimp

The overall prevalence of *Vibrio* spp. in retail shrimp samples in this study was 60.25% (241/400). Farmed samples (78.44%, 211/269) had higher *Vibrio* prevalence than wild caught samples (45.80%, 60/131). *Vibrio* prevalence was also higher in imported samples (71.61%, 227/317) than domestic samples (53.01%, 44/83). *Enterococcus* spp. were present in 89.75% of all samples (359/400), including 92.94% of farmed samples (250/269), 83.21% of wild caught samples (109/131), 91.17% of imported samples (289/317), and 84.34% of domestically sourced samples (70/83) (Table 1).

**Table 1:** Prevalence of *Vibrio* spp. and *Enterococcus* spp. in retail shrimp samples.

Variable	*Vibrio* spp. prevalence % (*n*/*N*)	*Enterococcus* spp. prevalence % (*n*/*N*)
Production type		
Wild caught	22.00% (53/241)	30.36% (109/359)
Farmed	78.00% (188/241)	69.64% (250/359)
Country of origin		
Argentina	2.49% (6/241)	6.41% (23/359)
Bangladesh	0.41% (1/241)	0.84% (3/359)
Canada	0.00% (0/241)	0.28% (1/359)
Ecuador	9.96% (24/241)	6.13% (22/359)
India	32.36% (78/241)	31.75% (114/359)
Indonesia	21.58% (52/241)	19.50% (70/359)
Mexico	4.56% (11/241)	5.01% (18/359)
Saudi Arabia	1.24%(3/241)	0.56% (2/359)
Thailand	7.88% (19/241)	5.57% (20/359)
U.S.	16.60% (40/241)	19.50% (70/359)
Vietnam	2.48% (6/241)	4.18% (15/359)
Not specified	0.41% (1/241)	0.28% (1/359)
Product source		
Domestic	16.60% (40/241)	19.50% (70/359)
Imported	83.40% (201/241)	47.63% (171/359)
Month of sample purchase		
Sep 2019	10.37% (25/241)	10.58% (38/359)
Oct 2019	15.76% (38/241)	15.04% (54/359)
Nov 2019	11.62% (28/241)	12.53% (45/359)
Dec 2019	14.52% (35/241)	12.53% (45/359)
Jan 2020	9.96% (24/241)	8.08% (29/359)
Feb 2020	7.47% (18/241)	5.85% (21/359)
May 2020	19.92% (48/241)	24.51% (88/359)
Jun 2020	10.37% (25/241)	10.86% (39/359)
Total	60.25% (241/400)	89.75% (359/400)

### 3.2. Phenotypic resistance from antimicrobial susceptibility testing

The predominant antimicrobials that the 110 *Vibrio* isolates tested for phenotypic resistance were resistant to were ampicillin (47.27%, 52/110) and cefoxitin (35.45%, 39/110). Low prevalence of resistance was observed for tetracycline (9.09%, 10/110), trimethoprim-sulfamethoxazole (8.18%, 9/110), amoxicillin/clavulanic acid 2:1 ratio (2.73%, 3/110), gentamicin (1.82%, 2/110), ciprofloxacin (0.91%, 1/110), and azithromycin (0%, 0/110) (Table 2). The number of *Vibrio* isolates with MIC values below the lowest concentration and above the highest concentration tested for those drugs in the Gram-negative panel are reported in Supplementary Table 2. Multidrug resistance was observed in 8.18% (9/110) of *Vibrio* isolates. A further 21.82% (24/110) were resistant to two antimicrobial classes, 35.45% (39/110) were resistant to one class, and the remaining 34.55% (38/110) were pansusceptible (Table 3).

**Table 2:** Distribution of *Vibrio* isolates resistant to antimicrobial agents.

Antimicrobial class	Antimicrobial agent	Number of resistant isolates	*Vibrio* resistance (%)
Aminoglycoside	Gentamicin	2	1.82
	Streptomycin	*	*
Phenicol	Chloramphenicol	*	*
Beta-lactam	Amoxicillin/clavulanic acid 2:1 ratio	3	2.73
Cephem	Cefoxitin	39	35.45
	Ceftriaxone	*	*
	Ceftiofur	*	*
Folate pathway antagonist	Sulfisoxazole	*	*
	Trimethoprim/sulfamethoxazole	9	8.18
Macrolide	Azithromycin	0	0
Penicillin	Ampicillin	52	47.27
Quinolone	Ciprofloxacin	1	0.91
	Nalidixic Acid	*	*
Tetracycline	Tetracycline	10	9.09

* Drugs for which resistance could not be determined due to lack of breakpoints.

**Table 3:** Distribution of phenotypic resistant patterns of *Vibrio* isolates (*n* = 110).

Resistance Pattern	No. of isolates with pattern n/N (%)	Drug classes
AMP	21/110 (19.09%)	Penicillins
AMP-FOX	20/110 (18.18%)	Cephems, penicillins
FOX	11/110 (10.00%)	Cephems
TET	6/110 (5.45%)	Tetracyclines
FOX-SXT-AMP*	4/110 (3.64%)	Cephems, penicillins, folate pathway antagonists
AMP-AUG2-FOX-SXT*	1/110 (0.91%)	Beta-lactams, cephems, penicillins, folate pathway antagonists
AMP-AUG2-FOX-GEN*	1/110 (0.91%)	Aminoglycosides, beta-lactams, cephems, penicillins
AMP-AUG2-FOX*	1/110 (0.91%)	Beta-lactams, cephems, penicillins
AMP-SXT-GEN*	1/110 (0.91%)	Aminoglycosides, penicillins, folate pathway antagonists
AMP-SXT-TET*	1/110 (0.91%)	Penicillins, folate pathway antagonists, tetracyclines
AMP-SXT	1/110 (0.91%)	Penicillins, folate pathway antagonists
AMP-TET	1/110 (0.91%)	Penicillins
CIP-TET	1/110 (0.91%)	Quinolones, tetracyclines
FOX-TET	1/110 (0.91%)	Cephems, tetracyclines
SXT	1/110 (0.91%)	Folate pathway antagonists
Pansusceptible	38/110 (34.55%)	–

* Patterns indicating multidrug resistance (resistance to three or more antimicrobial classes).

GEN, gentamicin; STR, streptomycin; AUG2, amoxicillin/clavulanic acid 2:1 ratio; FOX, cefoxitin; AXO, ceftriaxone; XNL, ceftiofur; SXT, trimethoprim/sulfamethoxazole; AZI, azithromycin; AMP, ampicillin; CHL, chloramphenicol; CIP, ciprofloxacin; NAL, nalidixic acid; TET, tetracycline.

Antimicrobial susceptibility testing of *Enterococcus* isolates revealed high prevalence of resistance to lincomycin (96.36%, 106/110), quinupristin-dalfopristin (87.27%, 96/110), ciprofloxacin (84.55%, 93/110), linezolid (78.18%, 86/110), erythromycin (52.73%, 58/110), and chloramphenicol (39.09%, 43/110) (Table 4). Only one isolate was pansusceptible to all drugs in the MIC panel and all other isolates exhibited resistance to at least one of these six drugs in addition to various combinations of the other drugs in the panel. Low levels of resistance were found for tetracycline (15.45%, 17/110), tylosin tartrate (13.64%, 15/110), nitrofurantoin (9.09%, 10/110), gentamicin (2.73%, 3/110), tigecycline (1.82%, 2/110), kanamycin (0.91%, 1/110), penicillin (0.91%, 1/110), vancomycin (0.91%, 1/110), daptomycin (0%, 0/110), and streptomycin (0%, 0/110). Of these *Enterococcus* isolates, 93.64% (103/110) were multidrug resistant, 3.64% (4/110) were resistant to two classes of antimicrobials, 1.82% (2/110) were resistant to one class, and 0.91% (1/110) were pansusceptible. The phenotypic resistance patterns of *Enterococcus* isolates were diverse, though half exhibited one of four patterns involving chloramphenicol (CHL), ciprofloxacin (CIP), erythromycin (ERY), lincomycin (LIN), linezolid (LZD), and quinupristin-dalfopristin (SYN): CIP-ERY-LIN-LZD-SYN (14.55%, 16/110), CIP-LIN-LZD-SYN (14.55%, 16/110), CHL-CIP-ERY-LIN-LZD-SYN (12.73%, 14/110), and CHL-CIP-LIN-LZD-SYN (8.18%, 9/110) (Table 5).

**Table 4:** Distribution of *Enterococcus* isolates resistant to antimicrobial agents.

Antimicrobial class	Antimicrobial agent	Number of resistant isolates	*Enterococcus* resistance (%)
Aminoglycoside	Streptomycin	0	0
	Kanamycin	1	0.91
	Gentamicin	3	2.73
Phenicol	Chloramphenicol	43	39.09
Glycopeptide	Vancomycin	1	0.91
Lincosamide	Lincomycin	106	96.36
Lipopeptide	Daptomycin	0	0
Macrolide	Tylosin tartrate	15	13.64
	Erythromycin	58	52.73
Nitrofuran	Nitrofurantoin	10	9.09
Oxazolidinone	Linezolid	86	78.18
Penicillin	Penicillin	1	0.91
Quinolone	Ciprofloxacin	93	84.55
Streptogramin	Quinupristin-dalfopristin	96	87.27
Tetracycline	Tigecycline	2	1.82
	Tetracycline	17	15.45

**Table 5:** Distribution of phenotypic resistant patterns of *Enterococcus* isolates (*n* = 110).

Resistance pattern	No. of isolates with pattern n/N (%)	Drug classes
CIP-ERY-LIN-LZD-SYN*	16/110 (14.55%)	Macrolides, lincosamides, oxazolidinones, quinolones, streptogramins
CIP-LIN-LZD-SYN*	16/110 (14.55%)	Lincosamides, oxazolidinones, quinolones, streptogramins
CHL-CIP-ERY-LIN-LZD-SYN*	14/110 (12.73%)	Lincosamides, macrolides, oxazolidinones, phenicols, quinolones, streptogramins
CHL-CIP-LIN-LZD-SYN*	9/110 (8.18%)	Lincosamides, oxazolidinones, phenicols, quinolones, streptogramins
CHL-CIP-ERY-LIN-LZD-SYN-TYLT*	3/110 (2.72%)	Lincosamides, macrolides, oxazolidinones, phenicols, quinolones, streptogramins
CHL-CIP-ERY-LIN-SYN*	3/110 (2.72%)	Lincosamides, macrolides, phenicols, quinolones, streptogramins
CIP-ERY-LIN-SYN*	3/110 (2.72%)	Lincosamides, quinolones, streptogramins
CIP-LIN-SYN*	3/110 (2.72%)	Lincosamides, quinolones, streptogramins
CHL-CIP-ERY-LIN-LZD-TET*	2/110 (1.81%)	Lincosamides, macrolides, oxazolidinones, phenicols, quinolones, tetracyclines
CIP-ERY-LIN-LZD-SYN-TYLT*	2/110 (1.81%)	Lincosamides, macrolides, oxazolidinones, quinolones, streptogramins
CHL-ERY-LIN-LZD-SYN-TET*	2/110 (1.81%)	Lincosamides, macrolides, oxazolidinones, phenicols, streptogramins, tetracyclines
CIP-LIN-LZD-SYN-TET*	2/110 (1.81%)	Lincosamides, oxazolidinones, quinolones, streptogramins, tetracyclines
ERY-LIN-LZD-SYN*	2/110 (1.81%)	Lincosamides, macrolides, oxazolidinones, streptogramins
CHL-CIP-LIN-NIT*	2/110 (1.81%)	Lincosamides, nitrofurans, phenicols, quinolones
LIN-LZD-SYN*	2/110 (1.81%)	Lincosamides, oxazolidinones, streptogramins
LIN-TET	2/110 (1.81%)	Lincosamides, tetracyclines
CHL-CIP-ERY-GEN-KAN-LIN-NIT-SYN-TET-TYLT*	1/110 (0.91%)	Aminoglycosides, lincosamides, macrolides, nitrofurans, phenicols, quinolones, streptogramins, tetracyclines
CHL-CIP-ERY-LIN-LZD-SYN-TET-TGC*	1/110 (0.91%)	Lincosamides, macrolides, oxazolidinones, phenicols, quinolones, streptogramins, tetracyclines
CIP-ERY-LIN-LZD-NIT-SYN-TET-TYLT*	1/110 (0.91%)	Lincosamides, macrolides, nitrofurans, oxazolidinones, quinolones, streptogramins, tetracyclines
CHL-CIP-ERY-LIN-LZD-SYN-TGC*	1/110 (0.91%)	Lincosamides, macrolides, oxazolidinones, phenicols, quinolones, streptogramins, tetracyclines
CHL-CIP-GEN-LIN-LZD-SYN-TYLT*	1/110 (0.91%)	Aminoglycosides, lincosamides, macrolides, oxazolidinones, phenicols, quinolones, streptogramins
CHL-CIP-LIN-LZD-SYN-TET*	1/110 (0.91%)	Lincosamides, oxazolidinones, phenicols, quinolones, streptogramins, tetracyclines
CHL-CIP-ERY-LIN-SYN-TYLT*	1/110 (0.91%)	Lincosamides, macrolides, phenicols, quinolones, streptogramins
CHL-CIP-LIN-LZD-SYN-TYLT*	1/110 (0.91%)	Lincosamides, macrolides, oxazolidinones, phenicols, quinolones, streptogramins
CHL-LIN-LZD-NIT-SYN-TYLT*	1/110 (0.91%)	Lincosamides, macrolides, nitrofurans, oxazolidinones, phenicols, streptogramins
CIP-ERY-LIN-LZD-NIT-SYN*	1/110 (0.91%)	Lincosamides, macrolides, oxazolidinones, quinolones, streptogramins
CIP-GEN-LIN-NIT-TET*	1/110 (0.91%)	Aminoglycosides, lincosamides, nitrofurans, quinolones, tetracyclines
CIP-LIN-LZD-SYN-TET*	1/110 (0.91%)	Lincosamides, oxazolidinones, quinolones, streptogramins, tetracyclines
CIP-LIN-LZD-SYN-TYLT*	1/110 (0.91%)	Lincosamides, macrolides, oxazolidinones, quinolones, streptogramins
CIP-LIN-NIT-TET-VAN*	1/110 (0.91%)	Glycopeptides, lincosamides, nitrofurans, quinolones, tetracyclines
ERY-LIN-LZD-SYN-TYLT*	1/110 (0.91%)	Lincosamides, macrolides, oxazolidinones, streptogramins
ERY-LIN-NIT-TET-TYLT*	1/110 (0.91%)	Lincosamides, macrolides, nitrofurans, tetracyclines
LIN-LZD-SYN-TET-TYLT*	1/110 (0.91%)	Lincosamides, macrolides, oxazolidinones, tetracyclines
CIP-ERY-LZD-SYN*	1/110 (0.91%)	Macrolides, oxazolidinones, quinolones, streptogramins
ERY-LZD-PEN-SYN*	1/110 (0.91%)	Macrolides, oxazolidinones, penicillins, streptogramins
LIN-LZD-NIT-SYN*	1/110 (0.91%)	Lincosamides, nitrofurans, oxazolidinones, streptogramins
CIP-LIN-NIT*	1/110 (0.91%)	Lincosamides, nitrofurans, quinolones
CIP-LIN-STR*	1/110 (0.91%)	Aminoglycosides, lincosamides, quinolones
CIP-LIN	1/110 (0.91%)	Lincosamides, quinolones
LIN-LZD	1/110 (0.91%)	Lincosamides, oxazolidinones
CIP	1/110 (0.91%)	Quinolones
LIN	1/110 (0.91%)	Lincosamides
Pansusceptible	1/110 (0.91%)	–

* Patterns indicating multidrug resistance (resistance to three or more antimicrobial classes).

### 3.3. Analysis of antimicrobial resistance by shrimp sample metadata

For analysis, origin was collapsed to domestic or imported categories due to small sample size by country. Similarly, season of collection was excluded due to small sample sizes by season. Packaging claims were excluded because few samples included claims about antimicrobial use. All claims that were found came in the form of the Global Seafood Alliance’s Best Aquaculture Practices (BAP) certifications, which mandate veterinary and regulatory oversight of antimicrobial usage and prohibit the use of drugs for growth promotion (Best Aquaculture Practices, 2014). The eight farmed *Vibrio* isolates from packages with BAP certifications averaged resistance to 1.88 drugs, which was more (*p* = 0.015) than the 0.96 average of the 73 isolates without the certification. Thirteen *Enterococcus* isolates were sourced from farmed shrimp samples with BAP certifications, and their average resistance to 5.31 drugs did not significantly differ (*p* = 0.212) from the average of the 4.79 drug average for the 68 uncertified. Shrimp species was excluded as a variable for analysis because the majority of samples were whiteleg shrimp (57.0%, 228/400) or did not specify species (21.75%, 87/400), and the remainder consisted of nine different species, which limited the ability to make comparisons between samples.

No significant associations were observed between multidrug resistance in *Vibrio* or *Entercoccus* isolates and production method (farm raised or wild caught) (Table 6). *Vibrio* isolates from farm raised shrimp were multidrug resistant 8.64% (7/81) of the time, compared to 6.90% (2/29) for those sourced from wild caught shrimp (*p* = 1.0). Of *Enterococcus* isolates from farm raised shrimp samples, 95.1% (77/81) were resistant to at least one of the sixteen antimicrobial agents, compared to 89.66% (26/29) from wild caught shrimp samples (*p* = 0.377). No significant associations were observed between multidrug resistance and sample origin either (domestic or imported) (Table 6). Domestic and imported *Vibrio* isolates were multidrug resistant 5.26% (1/19) and 8.79% (8/91) of the time, respectively (*p* = 1.0). *Enterococcus* isolates from domestically produced shrimp were multidrug resistant 88.89% (16/18) of the time, while those from imported shrimp were multidrug resistant 94.57% (87/92) of the time (*p* = 0.321).

**Table 6:** Association between multidrug resistant (MDR) *Vibrio* and *Enterococcus* from retail shrimp and sample production method and origin.

		*Vibrio*		*Enterococcus*	
		No. MDR	No. not MDR	No. MDR	No. not MDR
Production method	Farm raised	7	74	77	4
	Wild caught	2	27	26	3
		*p* = *1.0*		*p* = 0.3773	
**Origin**	Domestic	1	18	16	2
	Imported	8	83	87	5
		*p* = 1.0		*p* = 0.3214	

### 3.4. *Vibrio* species identification and metadata trends via whole genome sequencing (WGS)

For the 42 *Vibrio* isolates that underwent whole genome sequencing, the distribution of species and resistance genes alongside metadata characteristics are summarized in Table 7. The most common *Vibrio* species identified by WGS was *V. metschnikovii* (24/42; 57.14%), followed by *V. parahaemolyticus* (12/42; 28.57%), *V. alginolyticus* (3/42; 7.14%), *V. cholerae* (2/42; 4.76%), and *V. fluvialis* (1/42; 2.33%). All seven domestic isolates subjected to WGS were speciated as *V. metschnikovii*, while the majority of imported isolates were either *V. metschnikovii* (17/35; 48.57%) or *V. parahaemolyticus* (12/35; 34.29%). The domestic isolates averaged 0.29 ARGs, compared to 1.63 ARGs on average for imported isolates (Table 8). While the WGS subsample included twice as many isolates sourced from farmed shrimp than wild caught shrimp, the bacterial species compositions within the groups were similar to each other and to the full sample selected for AST.

**Table 7:** Distribution and sample characteristics of *Vibrio* isolates (*n* = 42) by species.

Species	No. of isolates *n*/*N* (%)	Average no. ARGs	Wild caught (%)	Farmed (%)	Domestic (%)	Imported (%)
*V. metschnikovii*	24/42 (57.14%)	0.71	37.50	62.50	29.17	70.83
*V. parahaemolyticus*	12/42 (28.57%)	3.00	25.00	75.00	0.00	100.00
*V. alginolyticus*	3/42 (7.14%)	0.33	33.33	66.67	0.00	100.00
*V. cholerae*	2/42 (4.76%)	2.50	0.00	100.00	0.00	100.00
*V. fluvialis*	1/42 (2.38%)	0.00	100.00	0.00	0.00	100.00

**Table 8:** Distribution of ARG abundance and *Vibrio* species by production type and sample origin.

Production type/origin	No. of isolates *n*/*N* (%)	Average no. ARGs	*V. metschnikovii* (%)	*V. parahaemolyticus* (%)	*V. alginolyticus* (%)	*V. cholerae* (%)	*V. fluvialis* (%)
Farmed	28/42 (66.67%)	0.93	53.57	32.14	7.14	7.14	0.00
Wild caught	14/42 (33.33%)	2.36	64.29	21.43	7.14	0.00	7.14
Domestic	7/42 (16.67%)	0.29	100.00	0.00	0.00	0.00	0.00
Imported	35/42 (83.33%)	1.63	48.57	34.29	8.57	5.71	2.86

### 3.5. Resistance gene identification through WGS

**Figure 1:**
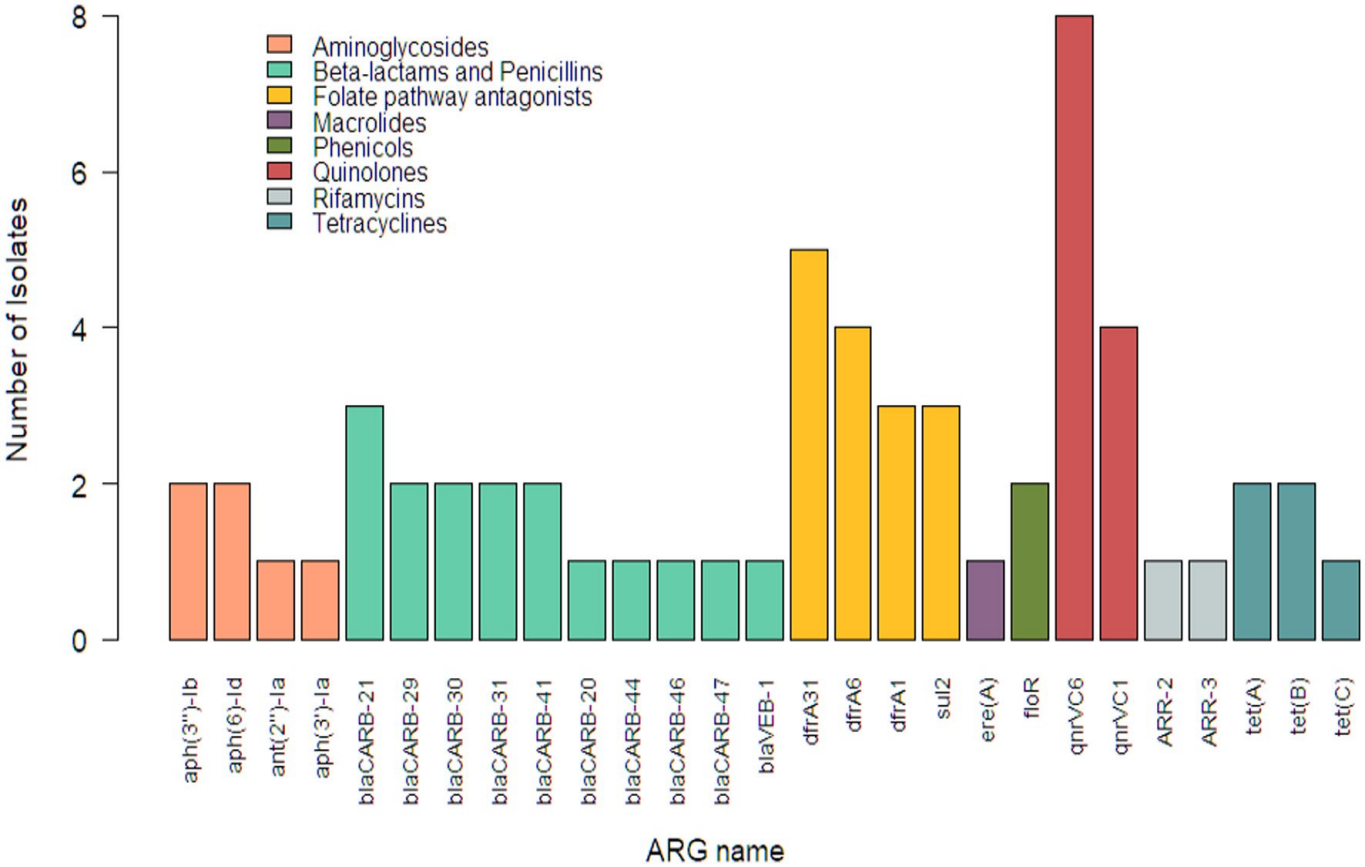
Distribution of antimicrobial resistance genes in *Vibrio* isolates (*n* = 42) from retail shrimp.

Whole genome sequencing identified 27 unique ARGs from the 42 *Vibrio* isolates. Among these resistance genes were genes corresponding to two types of aminoglycoside-modifying enzymes (AMEs), phosphotransferases (*aph(3′)-Ia*, *aph(3″)-Ib*, and *aph(6)-Id*) and adenylyltransferases (*aph(2″)-Ia*). Nine unique *bla*_CARB_ and one *bla*_VEB_ ARGs were found, which are associated with resistance to beta-lactam agents including penicillins. Four ARGs associated with folate pathway antagonists were identified, three of which (*dfr*A1, *dfr*A6, and *dfr*A31) are known to confer resistance to trimethoprim and one (*sul2*) known to confer resistance to sulfamethoxazole. Three ARGs encoding for tetracycline efflux pumps, two pentapeptide genes conferring resistance to quinolones, one chloramphenicol efflux pump gene, and one macrolide inactivation gene were also present in this subsample of *Vibrio* isolates. Two genes were identified that confer resistance to rifamycins, a drug class not included in the Gram-negative panel used for AST in this study. Cephems were the only class on the panel for which no ARGs were identified. The frequencies at which these ARGs were observed are visualized in Figure 1.

*Vibrio parahaemolyticus* isolates in this study had more ARGs than other species, averaging 3.00 ARGs per isolate. They were the only isolates to contain resistance genes associated with beta-lactams including penicillins. Sixteen unique resistance genes were identified in one *V. parahaemolyticus* isolate from a wild caught shrimp originating from Vietnam. This was also the only isolate with rifamycin and macrolide resistance genes and was one of only two with aminoglycoside resistance genes. All five ARGs identified from *V. metschnikovii* isolates corresponded to either quinolones or folate pathway antagonists.

**Figure 2:**
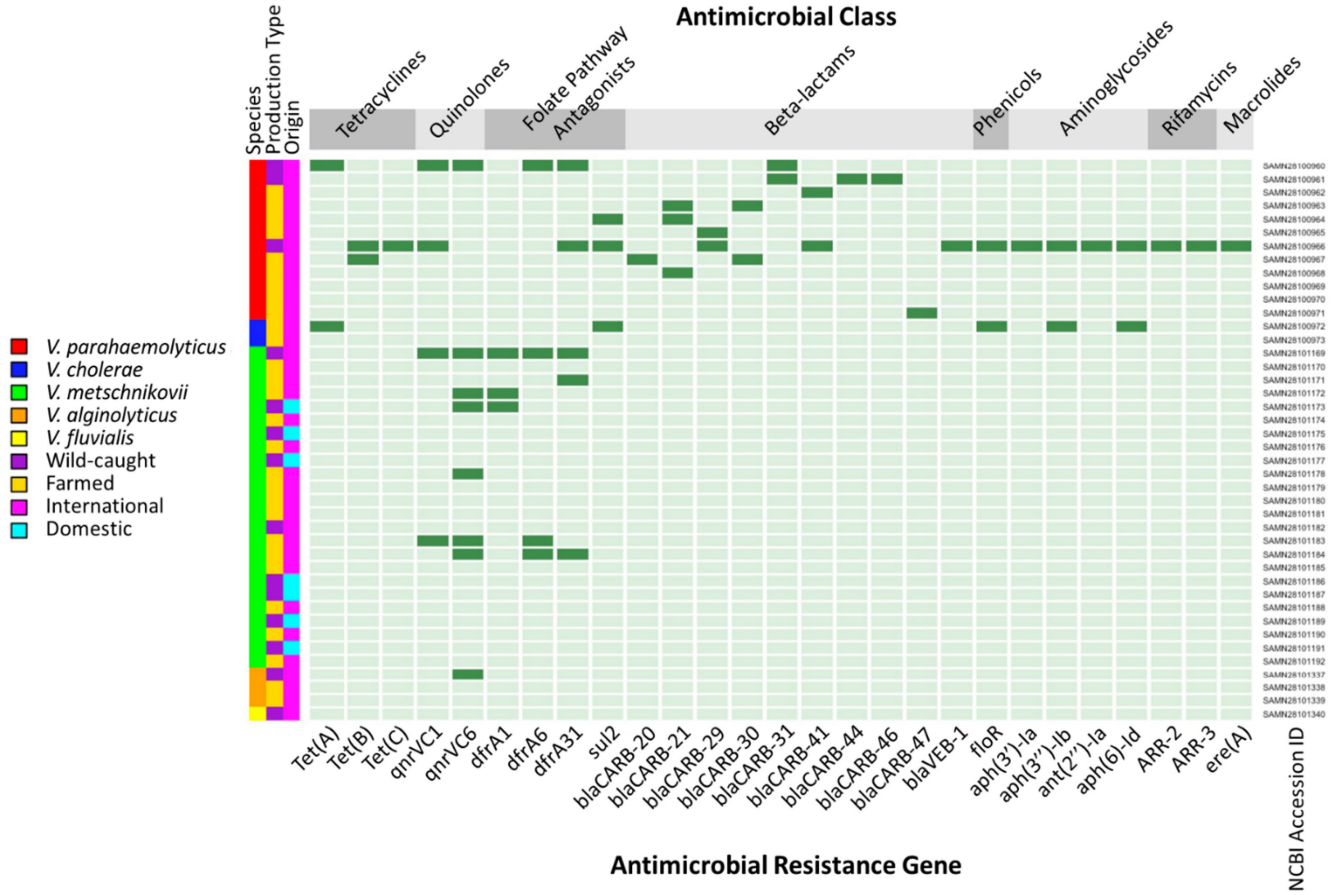
Heatmap of antimicrobial resistance genes (ARGs) identified through whole-genome sequencing in *Vibrio* isolates from retail shrimp. Dark green indicates the presence of an ARG and light green indicates the absence of an ARG.

Farmed isolates (28/42; 66.67%) contained between zero and five ARGs (mean = 0.93), while wild caught isolates (14/42; 33.33%) contained between zero and sixteen (mean = 2.36). The quinolone ARG *qnrVC6* was the predominant resistance gene identified in *Vibrio* isolates from farmed (4/28; 14.29%), wild caught (4/14; 28.57%), and imported shrimp. Only two ARGs were identified within the seven domestic isolates, *qnrVC6* (1/7; 14.29%) and *dfrA1* (1/7, 14.29%) (Figure 2).

### 3.6. Concordance of phenotypic and genotypic resistance in *Vibrio* isolates

Comparing phenotypic AMR and resistance genes identified from WGS in 40 non-cholera *Vibrio* isolates, the overall sensitivity and specificity were determined to be 11.54 and 96.05%, respectively. Discrepancies were observed in all drugs assessed; for each, there was at least one isolate that was categorized as phenotypically resistant but did not harbor any corresponding ARGs. None of the 17 isolates categorized as phenotypically resistant (3 resistant and 14 intermediate isolates) to cefoxitin harbored any associated ARGs. Of the four isolates categorized as phenotypically resistant (3 resistant and 1 intermediate) to trimethoprim/sulfamethoxazole, only one had ARGs associated with both component drugs. The majority of the false positives that contributed to low specificity were for ampicillin (ampicillin associated ARGs were found in 7 of the 15 phenotypically susceptible isolates) (Table 9).

**Table 9:** Concordance of phenotypic and genotypic resistance of non-cholera *Vibrio* isolates from retail shrimp (*n* = 40).

Antimicrobial class	Antimicrobial agent	Phenotypically susceptible (No. isolates)		Phenotypically resistant (No. isolates)		Sensitivity (%)^b^	Specificity (%)^c^
		Genotype: resistant (FP)^a^	Genotype: susceptible (TN)^a^	Genotype: resistant (TP)^a^	Genotype: susceptible (FN)^a^		
Aminoglycoside	GEN	1	37	0	2	0	97.37
Beta-lactam	AUG2	0	39	0	1	0	100
Cephem	FOX	0	23	0	17	0	100
Sulfonamide	SXT	0	36	1	3	25.00	100
Macrolide	AZI	0	40	0	0	N/A^d^	100
Penicillin	AMP	7	8	3	22	12.00	53.33
Tetracycline	TET	1	36	2	1	66.67	97.30
Overall		9	219	6	46	11.54	96.05

a FP, false positive; TN, true negative; TP, true positive; FN, false negative.

b Sensitivity was calculated as TP/(TP + FN).

c Specificity was calculated as TN/(TN + FP).

d Sensitivity could not be calculated for azithromycin due to lack of phenotypic resistance.

GEN, gentamicin; AUG2, amoxicillin/clavulanic acid 2:1 ratio; FOX, cefoxitin; SXT, trimethoprim/sulfamethoxazole; AZI, azithromycin; AMP, ampicillin; TET, tetracycline.

## 4. Discussion

### 4.1. Prevalence of *Vibrio* spp. and *Enterococcus* spp. in retail shrimp

This study found *Vibrio* prevalence of 60.25% (241/400) in retail shrimp meat samples of different production types and geographic origins in northern California. Other studies from around the world have reported widely varying prevalences of *Vibrio* spp. in shrimp samples, ranging from 17.1% in Iran to 88.1% in Mexico and 95.6% in Ecuador (Sperling et al., 2015; Asgarpoor et al., 2018; Guardiola-Avila et al., 2020). The most directly comparable recent assessment to ours was conducted in 2022 by Tate et al. who found 40.85% (290/710) prevalence of *Vibrio* spp. in United States retail shrimp samples. In this context, the 60.25% *Vibrio* spp. prevalence we found in our 400 retail shrimp samples is not anomalous.

We observed *Enterococcus* spp. prevalence to be 89.75% (359/400) in our shrimp samples. *Enterococcus* spp. are ubiquitous bacteria common in aquatic environments and the overall prevalence in our samples, while high, was in line with expectations. Our prevalence observations were similar to those of other recent studies that measured *Enterococcus* spp. in samples of retail shrimp meat, which ranged from 58.33% prevalence in shrimp imported to grocery stores in northeastern Poland, to 66% in shrimp samples from American grocery stores, to 84.7% in shrimp imported to Denmark (Chajęcka-Wierzchowska et al., 2016; Ellis-Iversen et al., 2020; Tate et al., 2022).

Whole genome sequencing is a powerful tool in both laboratory and clinical settings and has become popular in the surveillance of foodborne pathogens for its utility in identifying the microbial species present in samples (Grundmann, 2014; Köser et al., 2014). In this study we employed WGS to determine the species of a subset of 42 *Vibrio* isolates and identify the diversity of ARGs within their genomes. While *V. cholerae*, as some strains are causative agents of epidemic cholera, and *V. parahaemolyticus* and *V. alginolyticus*, as two of the most common causative agents of foodborne illness globally, are the species most associated with human infections that were found among our isolates, all of the species identified are among the twelve known to be associated with human infections (Morris and Acheson, 2003). The most prevalent *Vibrio* species we isolated was *V. metschnikovii* (57.14%; 24/42), followed by *V. parahaemolyticus* (28.57%; 12/42), *V. alginolyticus* (7.14%; 3/42), *V. cholerae* (4.76%; 2/42), and *V. fluvialis* (2.38%; 1/42). By comparison, in a 2011 assessment of *Vibrio* prevalences in shrimp samples in Baton Rouge, Louisiana, U.S., Wang et al. (2011) observed *V. cholerae* in 17.8%, *V. mimicus* in 6.63%, *V. parahaemolyticus* in 4.57%, and other, unspecified *Vibrio* species in 21.1% of their samples.

### 4.2. Phenotypic resistance of *Vibrio* and *Enterococcus*

The evaluation of *Vibrio* resistance was hindered by six drugs without defined CLSI or NARMS breakpoints on the 14-drug NARMS Gram-negative panel. One such drug was nalidixic acid, a quinolone. Quinolones are the most common class of antimicrobial agents used in aquaculture globally (Schar et al., 2020). Chloramphenicol also does not have defined breakpoints despite being highly relevant to aquaculture. It belongs to the third most commonly used class of antimicrobials in aquaculture globally, phenicols, and was the only drug in the panel approved for aquacultural use in the United States (Schar et al., 2020; FDA, 2022a).

The drugs that *Vibrio* isolates in this study were most commonly resistant to were ampicillin and cefoxitin, which is in line with previous assessments. Ampicillin resistance has been reported in *V. parahaemolyticus* and *V. vulnificus* since 1978 and 2001, respectively (Joseph et al., 1978; Zanetti et al., 2001). The observed prevalence of *Vibrio* resistance to ampicillin in our study (40.91%; 45/110) is very similar to that observed by Akinbowale et al. (2006) in their analysis of *Vibrio* isolates collected from various aquacultural sources in Australia (40.32%, 25/62), but contrasts starkly with Raissy et al. (2012) who found 97.2% (70/72) ampicillin resistance among *Vibrio* isolates from wild caught seafood. The prevalence of cefoxitin resistant *Vibrio* isolates (35.45%) in our study was similar to the findings of García-Aljaro et al. (2014), who found 44% resistance in *Vibrio* spp. isolates from aquaculture facilities.

Antimicrobial susceptibility testing of *Enterococcus* isolates revealed resistance prevalence for multiple drugs, namely lincomycin (96.36%), quinupristin-dalfopristin (87.27%), ciprofloxacin (84.55%), linezolid (78.18%), erythromycin (52.73%), and chloramphenicol (39.09%). Interestingly, despite near-ubiquitous resistance to lincomycin among *Enterococcus* isolates in this assessment, its use in aquaculture has only been reported in China and none of our samples originated from China (Lulijwa et al., 2020). This could suggest exposure to lincomycin residues from non-aquaculture sources; lincomycin is commonly found in the waste streams of terrestrial livestock facilities and could enter surface waterways that feed directly into flow-through aquaculture systems like those that predominate shrimp farming (Boyd et al., 2022; Public Health Agency of Canada, 2022).

Co-selection of AMR, a phenomenon in which selective pressure upon exposure to one antimicrobial agent often results in the acquisition of resistance to other agents, could also have contributed to the high prevalence of lincomycin resistance (Seiler and Berendonk, 2012; Zhang M et al., 2018; Imran et al., 2019). Some heavy metals, notably copper sourced from aquacultural and agricultural pollution, have been shown to cause co-selection of AMR in waterborne bacteria. Seiler and Berendonk (2012) found that exposure to high levels of copper resulted in bacterial resistance to lincomycin, erythromycin, and vancomycin which persisted through the end of their seven-day observation period. It’s possible that the high rates of resistance to lincomycin observed in our assessment could have resulted from inadvertent exposure to metals such as copper.

It is also possible that the use of lincomycin in the countries of origin for these resistant isolates was unreported, or even inadvertent. Accurate tracking of antimicrobial use is a difficult endeavor that often involves non-governmental surveys which depend on the honesty and knowledge of producers, both of which can be unreliable (Shamsuzzaman and Biswas, 2012; Pham et al., 2015). Mislabeling of probiotic products in shrimp and other aquaculture has also introduced unintended and antimicrobial resistant bacteria into farms, which could be another explanation for the observed lincomycin resistance (Noor Uddin et al., 2015; Uma and Rebecca, 2018).

Ciprofloxacin is not used in aquaculture, yet many of our *Enterococcus* isolates from both farm raised and wild caught shrimp grew uninhibited in its presence (Thornber et al., 2020). Other studies have reported varied levels of ciprofloxacin resistance. Ellis-Iversen et al. (2020) found near-zero resistance to ciprofloxacin in *E. faecalis* and *E. faecium* isolates from Asian seafood imported to Denmark, while Igbinosa and Beshiru (2019) found more than 40% ciprofloxacin resistance in *Enterococcus* isolated from ready-to-eat seafood products. The prevalence observed in our study (84.55%), however, is abnormally high compared to levels of ciprofloxacin resistance in previous studies. Ciprofloxacin is classified as a critically important antimicrobial in human medicine by the World Health Organization (WHO), who cite its frequency of use and unique effectiveness against pathogenic infections as reasons that resistance could pose a significant risk to human health (WHO et al., 2019). While *Enterococcus* spp. are not among the pathogens of concern in this case, populations with prevalent resistance like those we observed are concerning as potential reservoirs of ARGs that could transfer to more significant pathogens.

Resistance of *Enterococcus* to linezolid has become increasingly common within the past decade, which is a growing concern in human medicine since it is used as a last resort treatment against vancomycin-resistant *Enterococcus* infections (Klare et al., 2015). Clinical studies have reported an increase in the rate of linezolid resistance among *E. faecium* samples over time, including <1% in 2008, >9% in 2014, and >20% in 2021 (Klare et al., 2015 Ma et al., 2021). Although the isolates in this assessment are not human pathogenic *E. faecalis*, the fact that 78.18% of them exhibited resistance to lincomycin and all have the capability of spreading that trait is notable. The pervasiveness of linezolid resistance in our assessment is also curious because the drug is not applied in aquaculture settings (Lulijwa et al., 2020). It is possible that this resistance could also have been acquired in a co-selection process. Pervasive, acquired resistance to a wide range of antimicrobial drugs in bacteria as adept at ARG transfer as *Enterococcus* poses a threat to human health.

Intrinsic resistance is a consideration when interpreting *Enterococcus* MIC results as well. Many *Enterococci* are known to be intrinsically resistant to aminoglycosides, although few samples were classified as resistant to streptomycin (1.82%), kanamycin (0.91%), or gentamicin (2.73%) in our susceptibility testing (Morrison et al., 1997; Harakeh et al., 2006). Some *Enterococcus* species also have unique resistances; *E. faecalis* is intrinsically resistant to streptogramins like quinupristin-dalfopristin, for example, and *E. gallinarum* and *E. casseliflavus* are intrinsically resistant to vancomycin (Morrison et al., 1997; Arias and Murray, 2012; Ellis-Iversen et al., 2020). These species-specific traits could not be considered in our assessment, however, since no *Enterococcus* isolates were identified beyond genus level.

### 4.3. Multidrug resistance patterns in *Vibrio* and *Enterococcus*

Prevalence of MDR in aquaculture-sourced *Vibrio* has increased in the 21st century (Han et al., 2007; Baker-Austin et al., 2009; Raissy et al., 2012; Shaw et al., 2014; Igbinosa, 2016). However, recent studies have varied substantially in their MDR observations. The prevalence of MDR for our *Vibrio* isolates (8.18%) was similar to the rates observed in aquacultured shrimp-associated bacteria by Singh et al. (2018) in their study in Punjab, India (8.4%; 10/119), and Helena Rebouças et al. (2011) conducted in northeastern Brazil (12.9%; 4/31). By contrast, Costa et al. (2015b) found that none of their 100 *Virbio* isolates from farmed shrimp in Brazil were resistant to three or more of the nine drug classes in their MIC panel, while a 2016 analysis of *Vibrio* spp. sampled from Nigerian aquaculture farms (Igbinosa, 2016) found that 57.49% of isolates (96/167) were resistant to at least three of the eight classes of antimicrobial drugs they tested. While these comparisons are valuable to contextualize the results of this assessment, it should be noted that there is variation in the composition of the drug panels between studies. The studies referenced above feature a similar number and identity of drugs and drug classes to those of our assessment, however. Moreover, the resolution of our findings was hampered by the omission of six drugs from the Gram-negative panel; many other studies assessing *Vibrio* spp. included multiple drugs within one or more classes, whereas our panel had only one representative per class among interpretable drugs.

We observed 93.64% MDR among *Enterococcus* isolates. This was driven in large part by pervasive resistance to lincomycin, quinupristin-dalfopristin, ciprofloxacin, linezolid, erythromycin, and chloramphenicol. Other recent studies have reported similar rates of MDR driven by ubiquitous or near-ubiquitous resistance to a subset of drugs. Enany et al. (2022), for instance, found that all of their 72 aquaculture-sourced *Enterococcus* isolates were resistant to chloramphenicol, macrolides azithromycin, and erythromycin. Further, 91.6% (66/72) of their isolates were resistant to tetracycline, and all exhibited resistance or intermediate resistance to nitrofurantoin. Generally, there has been a growing trend of MDR *Enterococcus* which is a concern in clinical circles, and the results of this study reinforce that pattern (Klare et al., 2015).

### 4.4. Multidrug resistance of *Vibrio* and *Enterococcus* by shrimp production and origin

No significant association was found between multidrug resistance in isolates sourced from farm raised or wild caught shrimp for *Vibrio* (*p* = 1.0) or *Enterococcus* (*p* = 0.377). This result was unexpected because shrimp raised in a farm environment are likely to be directly exposed to antimicrobial drugs which would apply selective pressure and presumably result in higher prevalence of resistance. Antimicrobial agents including many of those included on the MIC panels in this assessment have increasingly been found at detectable concentrations in coastal and estuarine ecosystems where wild shrimp are fished, and even diffuse, subinhibitory concentrations have been shown to select for AMR in environmental and shrimp-associated bacteria (Gullberg et al., 2011; Zheng et al., 2021). One other possibility is that the wild caught samples as a group were contaminated between capture and sale in a way that farmed samples were not. Still, our results imply that wild caught shrimp do not pose lower risk than farm raised shrimp for AMR.

There was also no significant association between MDR in isolates sourced from domestically produced or imported shrimp for *Vibrio* (*p* = 1.0) or *Enterococcus* (*p* = 0.321). All domestic samples were labeled as wild caught at collection, but since no statistical difference was found between farmed and wild caught isolates, that should not affect the interpretation of this result. The similarity between samples of different geographic origins could indicate that the United States’ import monitoring has been successful in holding imported seafood to the same antimicrobial stewardship standards as domestic seafood, or that there is some overlap in the processing or distribution processes that facilitates cross-contamination of bacteria before all shrimp of any origin reach grocery store shelves (FDA, 2022b).

### 4.5. Distribution of antimicrobial resistance genes in *Vibrio* and concordance of phenotypic and genotypic antimicrobial resistance

Whole genome sequencing with a subset of 42 *Vibrio* isolates was performed after antimicrobial susceptibility testing to identify species and ARGs. The WGS revealed that two of the 42 isolates were *V. cholerae*, and it’s possible others among the non-sequenced isolates were as well. This complicates the interpretation of the results of the MIC analysis, because, as noted in results section 3.6, the breakpoints used to classify the MIC values were specifically defined for non-cholera *Vibrio*. The confirmed *V. cholerae* isolates were still considered in the MIC analysis since the identities of those not yet sequenced are unknown. If possible, sequencing isolates before MIC testing would help ensure that this uncertainty does not arise in future assessments. The most common ARGs found in this assessment were *qnr* genes which encode for pentapeptide repeat proteins and confer reduced susceptibility to quinolones. Resistance to quinolones is primarily mediated by chromosomal quinolone resistance determining region (QRDR) mutations, though, and secondarily by acquired plasmid-mediated quinolone resistance (PMQR) genes like *qnrVC1* and *qnrVC6* (Zhang Y et al., 2018; Esmaeel et al., 2020; Lee et al., 2022). Still, acquired ARGs impart partial resistance to quinolones on their own and remain dangerous since they are highly transmissible to other organisms and contribute to the selection of resistance-associated chromosomal mutations (Nazik et al., 2011). The raw MIC values for ciprofloxacin and nalidixic acid did not suggest that isolates with one or both of *qnrVC1* and *qnrVC6* had reduced phenotypic susceptibility to quinolones. Considering only 0.91% of *Vibrio* isolates were classified as resistant to ciprofloxacin, this likely indicates that few QRDR mutations were present in this subsample.

More unique ARGs were found related to beta-lactam and penicillin resistance than any other antimicrobial classes. Nine of the ten such genes were *bla_CARB_* ARGs. The beta-lactamase protein encoded by this class of genes is a major mechanism of resistance to beta-lactam agents in *Vibrio* spp. and beyond (Potron et al., 2009; Manjusha and Bhat, 2011; Li et al., 2020). The *bla_CARB_* ARGs identified in this study are almost exclusively found in *V. parahaemolyticus*; *bla_VEB-1_*, the other beta-lactamase ARG found in one of our isolates, was found in *V. parahaemolyticus* in this study but has also been previously observed in *V. alginolyticus* (Alcock et al., 2020).

Genotypic resistance to the trimethoprim/sulfamethoxazole combo agent would require ARGs for both drugs to be present, though only one still confers partial resistance (Suhartono et al., 2016; Das et al., 2020 Ma et al., 2021). Among the seven isolates with one or more allelic variants of the *dfrA* trimethoprim ARG without any sulfamethoxazole ARGs, there was a trend between number of unique variants and resistance to trimethoprim/sulfamethoxazole (SXT). Of the four isolates with three *dfrA* variants, two expressed phenotypic resistance. In addition, of the two isolates with two *dfrA* variants, one expressed intermediate resistance; the one isolate with one *dfrA* variant was phenotypically susceptible to SXT. Both isolates with sulfamethoxazole-associated *sul2* gene and no trimethoprim ARGs were susceptible to the combination agent. The other 32 isolates devoid of folate pathway antagonist ARGs were susceptible to SXT in MIC analysis.

An isolate with sixteen ARGs was the only with both a trimethoprim ARG (*dfr*A31) and a sulfamethoxazole ARG (*sul2*) and was phenotypically resistant to SXT. Among the other ARGs this isolate contained was aminoglycoside adenylyltransferase ARG *ant(2″)-Id*, which is known to confer resistance to gentamicin, though the isolate did not express phenotypic resistance to this drug in MIC testing (Ramirez and Tolmasky, 2010). Two aminoglycoside phosphotransferase ARGs, *aph(3″)-Ib* and *aph(6)-Id*, were also among the sixteen. These genes have been observed colocalized with *sul2* and other ARGs on RSF1010, an oft-transmitted plasmid (Ramirez and Tolmasky, 2010). Another isolate, an isolate speciated as *V. cholerae* from a farmed Ecuadorian shrimp, also contained *sul2*, *aph(3″)-Ib* and *aph(6)-Id*. Regardless of whether the RSF1010 plasmid is present in these isolates, the possibility highlights the high transfer potential of the ARGs identified in this study.

Tetracyclines are highly important agents for human and veterinary medicine (WHO et al., 2019). The tetracycline ARGs identified in this study are frequently found in bacterial genomes isolated from aquatic environments like aquaculture ponds and from crustaceans (Schmidt et al., 2001; Dang et al., 2007; Zhang et al., 2009; Kim et al., 2013). Their presence in this study means they could be spread to significant pathogens and complicate treatment in clinical settings.

Analyses of AMR should account for intrinsic resistances in the bacteria of interest (Michelle et al., 2016). It has been shown that *V. vulnificus* and *V. parahaemolyticus* are intrinsically resistant to cefoxitin (Elmahdi et al., 2016). The twelve *V. parahaemolyticus* isolates identified by our WGS, however, did not reflect this pattern: 11/12 (91.67%) were inhibited at cefoxitin concentrations low enough to classify them as susceptible, and the other one (8.33%) was classified as intermediate. Regardless, since not all 110 isolates subjected to antimicrobial susceptibility testing were sequenced, this would not have been accounted for in statistical analyses even if the intrinsic resistance was observed.

Chiou et al. (2015) posited that *V. parahaemolyticus* intrinsically carries the *bla_CARB-17_* gene which confers resistance to ampicillin, however only two of the twelve isolates identified in our study (16.67%) were phenotypically resistant to ampicillin and one (8.33%) expressed intermediate resistance. By contrast, among the other 28 non-cholera *Vibrio* isolates, 21 were phenotypically resistant (75.0%) and another was intermediate (3.57%). Further, WGS did not identify *bla_CARB-17_* in any of the 42 isolates, though nine other *bla_CARB_* genes were found in *V. parahaemolyticus* isolates.

We observed a trend in our study that *Vibrio* from wild caught shrimp harbored a higher number ARGs on average than those from farmed samples. This is an unexpected finding given that there was no significant difference in phenotypic resistance prevalence between these two groups. Domestic isolates in this subset averaged fewer ARGs (0.29) than imported isolates (1.63), though the sample sizes and species compositions of these subgroups were distinct and limited the utility of their comparison. All seven domestic isolates were speciated as *V. metschnikovii*, whereas the 35 imported isolates had a more representative species distribution. There was also no significant difference between these two groups in phenotypic resistance prevalence.

The identification of ARGs via WGS facilitates *in-silico* predictions of phenotypic resistance (NIHR Global Health Research Unit on Genomic Surveillance of AMR, 2020; Lee et al., 2022). Phenotypic and genotypic AMR in non-cholera *Vibrio* isolates in our study correlated with an overall sensitivity of 11.54% and specificity of 96.05%. There are a few explanations for this low sensitivity and imperfect specificity. The largest contributing factor to the low sensitivity in our study is likely the grouping of intermediate isolates with resistant isolates, particularly for cefoxitin where 14 intermediate isolates were categorized as resistant for analysis. The results from our dataset indicate that the treatment of intermediate isolates during analysis has a large impact on the assessment of phenotypic and genotypic concordance. Other explanations include our WGS analysis of ARGs being limited to one database, so it is possible there are undetected and/or unknown AMR genetic determinants present amongst our isolates, in addition to the potential impact of cut-offs for identity and coverage used to determine the presence of ARGs. Lastly, AST and WGS in our study were conducted on separate occasions, so it is possible that plasmid loss occurred at some point, which could further contribute to incongruence of phenotypic and genotypic AMR.

## 5. Conclusion

The large-scale production and global distribution demands for shrimp results in a food production system that can be conducive to the selection and spread of antimicrobial resistance, prompting the need to better understand the occurrence of AMR in both pathogenic and commensal bacteria from these products. This present study provides food safety and public health insights on the prevalence and distribution of AMR in *Vibrio* spp. and *Enterococcus* spp. from retail shrimp in California, and highlights the importance of continued AMR monitoring of seafood products and the value of complementing antimicrobial susceptibility testing with whole-genome sequencing for AMR assessment.

## Data availability statement

The datasets presented in this study can be found in online repositories. The names of the repository/repositories and accession number(s) can be found in the article/Supplementary material.

## Author contributions

XL, EA, XY, and KaL: conceptualization. BH, KuL, BB, MG, ZL, and AY: laboratory analysis. BH, KuL, KaL, DK, ZL, and AY: data curation. BH, KaL, DK, and XL: formal analysis. BH, KaL, DK, XL, and XY: methodology. KaL, XL, and XY: supervision. EA, DK, XL, and XY: resources. BH: writing – original draft preparation. KaL, DK, ZL, AY, XL, and XY: writing – review and editing. XL and EA: funding acquisition. All authors have read and agreed to the published version of the manuscript.

## Funding

This research was funded by USDA National Institute of Food and Agriculture Animal Health Formula Funds project no. CALV-AH-395.

## Conflict of interest

The authors declare that the research was conducted in the absence of any commercial or financial relationships that could be construed as a potential conflict of interest.

## Publisher’s note

All claims expressed in this article are solely those of the authors and do not necessarily represent those of their affiliated organizations, or those of the publisher, the editors and the reviewers. Any product that may be evaluated in this article, or claim that may be made by its manufacturer, is not guaranteed or endorsed by the publisher.

## Figures and Tables

**Table:** 

AMR	antimicrobial resistance
AST	antimicrobial susceptibility testing
WGS	whole genome sequencing
FAO	Food and Agriculture Organization of the United Nations
CDC	United States Centers for Disease Control and Prevention
NARMS	National Antimicrobial Resistance Monitoring System
FDA	United States Food and Drug Administration
APW	alkaline peptone water
BPW	buffered peptone water
TCBS	thiosulfate-citrate-bile salts-sucrose
OD	optical density
CAMHB	cation-adjusted Mueller-Hinton broth
CLSI	Clinical and Laboratory Standards Institute
MDR	multidrug resistance
ARG	antimicrobial resistance gene
TSA-YE	trypticase soy agar with 0.6% yeast extract
TP	true positive
TN	true negative
FP	false positive
FN	false negative
GEN	gentamicin
STR	streptomycin
AUG2	amoxicillin/clavulanic acid 2:1 ratio
FOX	cefoxitin
AXO	ceftriaxone
XNL	ceftiofur
SXT	trimethoprim/sulfamethoxazole
AZI	azithromycin
AMP	ampicillin
CHL	chloramphenicol
CIP	ciprofloxacin
NAL	nalidixic acid
TET	tetracycline
KAN	kanamycin
VAN	vancomycin
LIN	lincomycin
DAP	daptomycin
TYLT	tylosin tartrate
ERY	erythromycin
NIT	nitrofurantoin
LZD	linezolid
PEN	penicillin
SYN	quinupristin-dalfopristin
TGC	tigecycline
BAP	Best Aquaculture Practices
AME	aminoglycoside-modifying enzyme
WHO	World Health Organization
QRDR	quinolone resistance determining region
PMQR	plasmid-mediated quinolone resistance
NIHR	National Institute for Health and Care Research

## References

[ref1] Akinbowale O. Peng H. Barton M. D. (2006). Antimicrobial resistance in bacteria isolated from aquaculture sources in Australia. J. Appl. Microbiol. 100, 1103–1113. doi: 10.1111/j.1365-2672.2006.02812.x, PMID: 16630011

[ref2] Alcock B. P. Raphenya A. R. Lau T. T. Y. Tsang K. K. Bouchard M. Edalatmand A.. (2020). CARD 2020: antibiotic resistome surveillance with the comprehensive antibiotic resistance database. Nucleic Acids Res. 48, D517–D525. doi: 10.1093/nar/gkz935, PMID: 31665441 PMC7145624

[ref3] Ali H. Rahman M. M. Rico A. Jaman A. Basak S. K. Islam M. M.. (2018). An assessment of health management practices and occupational health hazards in tiger shrimp (*Penaeus monodon*) and freshwater prawn (*Macrobrachium rosenbergii*) aquaculture in Bangladesh. Vet Animal Sci. 5, 10–19. doi: 10.1016/j.vas.2018.01.002, PMID: 32734040 PMC7386765

[ref4] Amatul-Samahah M. A. Wan Omar W. H. H. Mohd Ikhsan N. F. Amal Azmai M. N. Zamri-Saad M. Ina-Salwany M. Y. (2020). Vaccination trials against vibriosis in shrimp: a review. Aquacult. Rep. 18:100471. doi: 10.1016/j.aqrep.2020.100471

[ref5] Arias C. A. Murray B. E. (2012). The rise of the *Enterococcus*: beyond vancomycin resistance. Nat. Rev. Microbiol. 10, 266–278. doi: 10.1038/nrmicro2761, PMID: 22421879 PMC3621121

[ref6] Aryal S. (2016). PYR test- principle, uses, procedure and result interpretation. Microbiology Info.com. Available at: https://microbiologyinfo.com/pyr-test-principle-uses-procedure-and-result-interpretation/ (Accessed February 20, 2023).

[ref7] Asgarpoor D. Haghi F. Zeighami H. (2018). Detection and molecular characterization of *Vibrio parahaemolyticus* in shrimp samples. Open Biotechnol. J. 12, 46–50. doi: 10.2174/1874070701812010046

[ref8] Baker-Austin C. McArthur J. V. Lindell A. H. Wright M. S. Tuckfield R. C. Gooch J.. (2009). Multi-site analysis reveals widespread antibiotic resistance in the marine pathogen *Vibrio vulnificus*. Microb. Ecol. 57, 151–159. doi: 10.1007/s00248-008-9413-8, PMID: 18642041

[ref9] Best Aquaculture Practices (2014). Aquaculture Facility Certification – finfish, crustacean, and mollusk hatcheries and nurseries – Version 1

[ref10] Bortolaia V. Kaas R. S. Ruppe E. Roberts M. C. Schwarz S. Cattoir V.. (2020). ResFinder 4.0 for predictions of phenotypes from genotypes. J. Antimicrob. Chemother. 75, 3491–3500. doi: 10.1093/jac/dkaa345, PMID: 32780112 PMC7662176

[ref11] Boyd C. E. Davis R. P. McNevin A. A. (2022). Perspectives on the mangrove conundrum, land use, and benefits of yield intensification in farmed shrimp production: a review. J. World Aquacult. Soc. 53, 8–46. doi: 10.1111/jwas.12841

[ref12] Byappanahalli M. N. Nevers M. B. Korajkic A. Staley Z. R. Harwood V. J. (2012). Enterococci in the environment. Microbiol. Mol. Biol. Rev. 76, 685–706. doi: 10.1128/MMBR.00023-12, PMID: 23204362 PMC3510518

[ref13] Çardak M. Özmen Toğay S. Ay M. Karaalioğlu O. Erol Ö. Bağcı U. (2022). Antibiotic resistance and virulence genes in *Enterococcus* species isolated from raw and processed seafood. J. Food Sci. Technol. 59, 2884–2893. doi: 10.1007/s13197-021-05313-z, PMID: 35734123 PMC9206942

[ref14] Centers for Disease Control and Prevention (CDC) (2019a). Antibiotic resistance threats in the United States, 2019. Atlanta, GA: U.S. Department of Health and Human Services, CDC

[ref15] Centers for Disease Control and Prevention (CDC) (2019b). Antibiotics Tested by NARMS. Available at: https://www.cdc.gov/narms/antibiotics-tested.html (Accessed July 14, 2022).

[ref16] Chajęcka-Wierzchowska W. Zadernowska A. Łaniewska-Trokenheim Ł. (2016). Virulence factors, antimicrobial resistance and biofilm formation in *Enterococcus* spp. isolated from retail shrimps. LWT Food Sci. Technol. 69, 117–122. doi: 10.1016/j.lwt.2016.01.034

[ref17] Chang Y.-H. Kumar R. Ng T. H. Wang H.-C. (2018). What vaccination studies tell us about immunological memory within the innate immune system of cultured shrimp and crayfish. Dev. Comp. Immunol. 80, 53–66. doi: 10.1016/j.dci.2017.03.003, PMID: 28279805

[ref18] Chiou J. Li R. Chen S. (2015). CARB-17 family of β-lactamases mediates intrinsic resistance to *Penicillins* in *Vibrio parahaemolyticus*. Antimicrob. Agents Chemother. 59, 3593–3595. doi: 10.1128/AAC.00047-15, PMID: 25801555 PMC4432138

[ref19] Clausen P. T. L. C. Aarestrup F. M. Lund O. (2018). Rapid and precise alignment of raw reads against redundant databases with KMA. BMC Bioinformat. 19:307. doi: 10.1186/s12859-018-2336-6, PMID: 30157759 PMC6116485

[ref20] Clinical and Laboratory Standards Institute (CLSI) (2017). Performance standards for antimicrobial susceptibility testing. CLSI supplement M100 27th Clinical Laboratory Standards Institute Wayne, PA, U.S.

[ref21] Clinical and Laboratory Standards Institute (CLSI) Methods for dilution antimicrobial susceptibility tests for bacteria that grow aerobically. CLSI document M07 11th (2018). Clinical Laboratory Standards Institute Wayne, PA, U.S.

[ref22] Costa R. A. Araújo R. L. dos Viera R. H. S. F. (2015a). Raw tropical oysters as vehicles for multidrug-resistant *Vibrio parahaemolyticus*. Rev. Inst. Med. Trop. Sao Paulo 57, 193–196. doi: 10.1590/S0036-46652015000300002, PMID: 26200957 PMC4544241

[ref23] Costa R. A. Araújo R. L. Souza O. V. dos Vieira R. H. S. F. (2015b). Antibiotic-resistant *Vibrios* in farmed shrimp. Biomed. Res. Int. 2015:e505914, 1–5. doi: 10.1155/2015/505914, PMID: 25918714 PMC4396125

[ref24] Dang H. Zhang X. Song L. Chang Y. Yang G. (2007). Molecular determination of oxytetracycline-resistant bacteria and their resistance genes from mariculture environments of China. J. Appl. Microbiol. 103, 2580–2592. doi: 10.1111/j.1365-2672.2007.03494.x, PMID: 18045442

[ref25] Das B. Verma J. Kumar P. Ghosh A. Ramamurthy T. (2020). Antibiotic resistance in *Vibrio cholerae*: understanding the ecology of resistance genes and mechanisms. Vaccine 38, A83–A92. doi: 10.1016/j.vaccine.2019.06.031, PMID: 31272870

[ref26] El-Far S. A. H. Khalil R. H. Saad T. T. Tanekhy M. E. Abdel-Latif H. M. R. (2015). Occurrence, characterization and antibiotic resistance patterns of bacterial communities encountered in mass kills of pond cultured Indian prawn (*Fenneropenaeus indicus*) at Damietta governorate, Egypt. Int. J. Fish. Aquat. Stud. 2, 271–276.

[ref27] Ellis-Iversen J. Seyfarth A. M. Korsgaard H. Bortolaia V. Munck N. Dalsgaard A. (2020). Antimicrobial resistant *E. coli* and enterococci in pangasius fillets and prawns in Danish retail imported from Asia. Food Control 114:106958. doi: 10.1016/j.foodcont.2019.106958

[ref28] Elmahdi S. DaSilva L. V. Parveen S. (2016). Antibiotic resistance of *Vibrio parahaemolyticus* and *Vibrio vulnificus* in various countries: a review. Food Microbiol. 57, 128–134. doi: 10.1016/j.fm.2016.02.008, PMID: 27052711

[ref29] Enany M. Tartor Y. H. Kishk R. M. Ali E. M. (2022). Occurrence of multidrug-resistant enterococci in fresh water fishes. Suez Canal Vet. Med. J. 27, 91–100. doi: 10.21608/scvmj.2022.125815.1073

[ref30] Esmaeel N. E. Gerges M. A. Hosny T. A. Ali A. R. Gebriel M. G. (2020). Detection of chromosomal and plasmid-mediated quinolone resistance among *Escherichia coli* isolated from urinary tract infection cases; Zagazig University Hospitals, Egypt. Infect. Drug Resist. 13, 413–421. doi: 10.2147/IDR.S240013, PMID: 32104013 PMC7023874

[ref31] FDA (2021). Seafood pilot study laboratory protocol. FDA National Antimicrobial Resistance Monitoring System Available at: https://www.fda.gov/media/149957/download

[ref32] FDA (2022a). Approved Aquaculture Drugs. Available at: https://www.fda.gov/animal-veterinary/aquaculture/approved-aquaculture-drugs (Accessed July 14, 2022).

[ref101] FDA (2022b). Imported Seafood Safety Program. Available at: https://www.fda.gov/food/importing-food-products-united-states/imported-seafood-safety-program (Accessed July 15, 2022).

[ref33] Food and Agriculture organization of the United Nations (FAO) (2022a). The state of the world fisheries and aquaculture: towards blue transformation Rome: Food and Agriculture Organization of the United Nations

[ref34] Food and Agriculture Organization of the United Nations (FAO) FishStatJ: global fishery and aquaculture production statistics Rome: Food and Agriculture Organization of the United Nations (2022b)

[ref35] García-Aljaro C. Riera-Heredia J. Blanch A. R. (2014). Antimicrobial resistance and presence of the SXT mobile element in *Vibrio* spp. isolated from aquaculture facilities. New Microbiol. 37, 339–346. PMID: 25180848

[ref36] Golder H. M. Séon Simon A. A. Santigosa E. de Ondarza M.-B. Lean I. J. (2022). Effects of probiotic interventions on production efficiency, survival rate, and immune responses of shrimp: a meta-analysis and meta-regression. Aquaculture 552:737973. doi: 10.1016/j.aquaculture.2022.737973

[ref37] Grundmann H. (2014). Towards a global antibiotic resistance surveillance system: a primer for a roadmap. Ups. J. Med. Sci. 119, 87–95. doi: 10.3109/03009734.2014.904458, PMID: 24694024 PMC4034565

[ref102] Guardiola‐Avila I. Martínez‐Vázquez V. Juárez‐Rendón K. Alvarez‐Ainza M. Paz‐González A. Rivera G. (2020). Prevalence and virulence of Vibrio species isolated from raw shrimp from retail markets in Reynosa, Mexico. Letters in Applied Microbiology. 71, 280–286. doi: 10.1111/lam.13315 32408383

[ref38] Gudding R. Van Muiswinkel W. B. (2013). A history of fish vaccination: science-based disease prevention in aquaculture. Fish Shellfish Immunol. 35, 1683–1688. doi: 10.1016/j.fsi.2013.09.031 24099805

[ref39] Gullberg E. Cao S. Berg O. G. Ilbäck C. Sandegren L. Hughes D.. (2011). Selection of resistant bacteria at very low antibiotic concentrations. PLoS Pathog. 7:e1002158. doi: 10.1371/journal.ppat.1002158, PMID: 21811410 PMC3141051

[ref40] Han F. Walker R. D. Janes M. E. Prinyawiwatkul W. Ge B. (2007). Antimicrobial susceptibilities of *Vibrio parahaemolyticus* and *Vibrio vulnificus* isolates from Louisiana Gulf and retail raw oysters. Appl. Environ. Microbiol. 73, 7096–7098. doi: 10.1128/AEM.01116-07, PMID: 17827331 PMC2074966

[ref41] Harakeh S. Yassine H. Hajjar S. El-Fadel M. (2006). Isolates of Staphylococcus aureus and saprophyticus resistant to antimicrobials isolated from the Lebanese aquatic environment. Mar. Pollut. Bull. 52, 912–919. doi: 10.1016/j.marpolbul.2005.12.008, PMID: 16487984

[ref42] Helena Rebouças R. Viana de Sousa O. Sousa Lima A. Roger Vasconcelos F. de Carvalho P. B. dos Fernandes Vieira R. H. S. (2011). Antimicrobial resistance profile of Vibrio species isolated from marine shrimp farming environments (*Litopenaeus vannamei*) at Ceará, Brazil. Environ. Res. 111, 21–24. doi: 10.1016/j.envres.2010.09.012, PMID: 20970784

[ref43] Holmström K. Gräslund S. Wahlström A. Poungshompoo S. Bengtsson B.-E. Kautsky N. (2003). Antibiotic use in shrimp farming and implications for environmental impacts and human health. Int. J. Food Sci. Technol. 38, 255–266. doi: 10.1046/j.1365-2621.2003.00671.x

[ref44] Igbinosa E. O. (2016). Detection and antimicrobial resistance of *Vibrio* isolates in aquaculture environments: implications for public health. Microb. Drug Resist. 22, 238–245. doi: 10.1089/mdr.2015.0169, PMID: 26540391

[ref45] Igbinosa E. O. Beshiru A. (2019). Antimicrobial resistance, virulence determinants, and biofilm formation of *Enterococcus* species from ready-to-eat seafood. Front. Microbiol. 10:728. doi: 10.3389/fmicb.2019.00728, PMID: 31057497 PMC6482160

[ref46] Imran M. Das K. R. Naik M. M. (2019). Co-selection of multi-antibiotic resistance in bacterial pathogens in metal and microplastic contaminated environments: an emerging health threat. Chemosphere 215, 846–857. doi: 10.1016/j.chemosphere.2018.10.114, PMID: 30359954

[ref47] Joseph S. W. DeBell R. M. Brown W. P. (1978). In vitro response to chloramphenicol, tetracycline, ampicillin, gentamicin, and beta-lactamase production by halophilic *Vibrios* from human and environmental sources. Antimicrob. Agents Chemother. 13, 244–248. doi: 10.1128/AAC.13.2.244, PMID: 646346 PMC352221

[ref48] Kim M. Kwon T.-H. Jung S.-M. Cho S.-H. Jin S. Y. Park N.-H.. (2013). Antibiotic resistance of bacteria isolated from the internal organs of edible snow crabs. PLoS One 8:e70887. doi: 10.1371/journal.pone.0070887, PMID: 23990916 PMC3749200

[ref49] Klare I. Fleige C. Geringer U. Thürmer A. Bender J. Mutters N. T.. (2015). Increased frequency of linezolid resistance among clinical *Enterococcus faecium* isolates from German hospital patients. J. Glob. Antimicrob. Resist. 3, 128–131. doi: 10.1016/j.jgar.2015.02.007, PMID: 27873661

[ref50] Köser C. U. Ellington M. J. Peacock S. J. (2014). Whole-genome sequencing to control antimicrobial resistance. Trends Genet. 30, 401–407. doi: 10.1016/j.tig.2014.07.003, PMID: 25096945 PMC4156311

[ref51] Kumar V. Roy S. Meena D. K. Sarkar U. K. (2016). Application of probiotics in shrimp aquaculture: importance, mechanisms of action, and methods of administration. Rev. Fish. Sci. Aquacult. 24, 342–368. doi: 10.1080/23308249.2016.1193841

[ref52] Lanz-Mendoza H. Contreras-Garduño J. (2022). Innate immune memory in invertebrates: concept and potential mechanisms. Develop. Comparat. Immunol. 127:104285. doi: 10.1016/j.dci.2021.104285, PMID: 34626688

[ref53] Lee K. Y. Atwill E. R. Pitesky M. Huang A. Lavelle K. Rickard M.. (2022). Antimicrobial resistance profiles of non-typhoidal *Salmonella* from retail meat products in California, 2018. Front. Microbiol. 13:835699. doi: 10.3389/fmicb.2022.835699, PMID: 35369434 PMC8966841

[ref54] Li P. Liu C. Li B. Ma Q. (2020). Structural analysis of the CARB β-lactamase from *Vibrio parahaemolyticus* facilitates application of the β-lactam/β-lactamase inhibitor therapy. Biochimie 171–172, 213–222. doi: 10.1016/j.biochi.2020.03.011, PMID: 32179166

[ref55] Lulijwa R. Rupia E. J. Alfaro A. C. (2020). Antibiotic use in aquaculture, policies and regulation, health and environmental risks: a review of the top 15 major producers. Rev. Aquac. 12, 640–663. doi: 10.1111/raq.12344

[ref56] Ma X. Zhang F. Bai B. Lin Z. Xu G. Chen Z.. (2021). Linezolid resistance in *Enterococcus faecalis* associated with urinary tract infections of patients in a tertiary hospitals in China: resistance mechanisms, virulence, and risk factors. Front. Public Health 9:570650. doi: 10.3389/fpubh.2021.570650, PMID: 33614576 PMC7893085

[ref57] Ma Q. Zhu C. Yao M. Yuan G. Sun Y. (2021). Correlation between the sulfamethoxazole-trimethoprim resistance of *Shigella flexneri* and the sul genes. Medicine 100:e24970. doi: 10.1097/MD.0000000000024970, PMID: 33725864 PMC7969299

[ref58] Manjusha S. Bhat S. (2011). Plasmid associated antibiotic resistance in *Vibrio* isolated from coastal waters of Kerala. Int. Food Res. J. 18, 1171–1181.

[ref59] Michelle K. Lihan S. Felecia C. Entigu R. Apun K. Bilung L. M.. (2016). Antibiotic resistance of diverse bacteria from aquaculture in Borneo. Int. J. Microbiol. 2016, 1–9. doi: 10.1155/2016/2164761, PMID: 27746817 PMC5055980

[ref60] Morris J. G. Acheson D. (2003). Cholera and other types of vibriosis: a story of human pandemics and oysters on the half shell. Clin. Infect. Dis. 37, 272–280. doi: 10.1086/375600, PMID: 12856219

[ref61] Morrison D. Woodford N. Cookson B. (1997). Enterococci as emerging pathogens of humans. J. Appl. Microbiol. 83, 89S–99S. doi: 10.1046/j.1365-2672.83.s1.10.x 28621900

[ref62] National Fisheries Institute Media (2022). Top 10 list offers a look back in time. National Fisheries Institute Media. Available at: https://aboutseafood.com/press_release/top-10-list-offers-a-look-back-in-time/ (Accessed May 16, 2022).

[ref63] National Marine Fisheries Service (2022). Fisheries of the United States, 2020. Available at: https://www.fisheries.noaa.gov/national/sustainable-fisheries/fisheries-united-states

[ref64] Nazik H. Bektöre B. Öngen B. Ilktaç M. Özyurt M. Kuvat N.. (2011). Plasmid-mediated quinolone resistance genes in *Escherichia coli* urinary isolates from two teaching hospitals in Turkey: coexistence of TEM, SHV, CTX-M and VEB-1 type lactamases. Trop. J. Pharm. Res. 10, 325–333. doi: 10.4314/tjpr.v10i3.9

[ref65] NIHR Global Health Research Unit on Genomic Surveillance of AMR (2020). Whole-genome sequencing as part of national and international surveillance programmes for antimicrobial resistance: a roadmap. BMJ Glob. Health 5:e002244. doi: 10.1136/bmjgh-2019-002244, PMID: 33239336 PMC7689591

[ref66] Noor Uddin G. M. Larsen M. H. Christensen H. Aarestrup F. M. Phu T. M. Dalsgaard A. (2015). Identification and antimicrobial resistance of bacteria isolated from probiotic products used in shrimp culture. PLoS One 10:e0132338. doi: 10.1371/journal.pone.0132338, PMID: 26147573 PMC4492959

[ref67] Pham D. K. Chu J. Do N. T. Brose F. Degand G. Delahaut P.. (2015). Monitoring antibiotic use and residue in freshwater aquaculture for domestic use in Vietnam. EcoHealth 12, 480–489. doi: 10.1007/s10393-014-1006-z, PMID: 25561382 PMC4623066

[ref68] Potron A. Poirel L. Croizé J. Chanteperdrix V. Nordmann P. (2009). Genetic and biochemical characterization of the first extended-Spectrum CARB-type ß-lactamase, RTG-4, from *Acinetobacter baumannii*. Antimicrob. Agents Chemother. 53, 3010–3016. doi: 10.1128/AAC.01164-08, PMID: 19380596 PMC2704689

[ref69] Public Health Agency of Canada (2022). Canadian Antimicrobial Resistance Surveillance System Report 2021 Available at: https://www.canada.ca/en/public-health/services/publications/drugs-health-products/canadian-antimicrobial-resistance-surveillance-system-report-2021.html (Accessed February 3, 2023).

[ref70] PulseNet (n.d.). Laboratory standard operating procedure for whole genome sequencing on MiSeq. Available at: https://www.cdc.gov/pulsenet/ (Accessed April 18, 2023).

[ref71] Raissy M. Moumeni M. Ansari M. Rahimi E. (2012). Antibiotic resistance pattern of some *Vibrio* strains isolated from seafood. Iran. J. Fish. Sci. 11, 618–626.

[ref72] Ramirez M. S. Tolmasky M. E. (2010). Aminoglycoside modifying enzymes. Drug Resist. Updat. 13, 151–171. doi: 10.1016/j.drup.2010.08.003, PMID: 20833577 PMC2992599

[ref73] Schar D. Klein E. Y. Laxminarayan R. Gilbert M. Van Boeckel T. P. (2020). Global trends in antimicrobial use in aquaculture. Sci. Rep. 10:21878. doi: 10.1038/s41598-020-78849-3, PMID: 33318576 PMC7736322

[ref74] Schmidt A. S. Brunn M. S. Dalsgaard I. Larsen J. L. (2001). Incidence, distribution, and spread of tetracycline resistance determinants and integron-associated antibiotic resistance genes among motile aeromonads from a fish farming environment. Appl. Environ. Microbiol. 67, 5675–5682. doi: 10.1128/AEM.67.12.5675-5682.2001, PMID: 11722922 PMC93359

[ref75] Seiler C. Berendonk T. (2012). Heavy metal driven co-selection of antibiotic resistance in soil and water bodies impacted by agriculture and aquaculture. Front. Microbiol. 3:399. doi: 10.3389/fmicb.2012.00399, PMID: 23248620 PMC3522115

[ref76] Shamsuzzaman M. Biswas T. K. (2012). Aqua chemicals in shrimp farm: a study from south-west coast of Bangladesh. Egypt. J. Aquat. Res. 38, 275–285. doi: 10.1016/j.ejar.2012.12.008

[ref77] Shaw K. S. Rosenberg Goldstein R. E. He X. Jacobs J. M. Crump B. C. Sapkota A. R. (2014). Antimicrobial susceptibility of *Vibrio vulnificus* and *Vibrio parahaemolyticus* recovered from recreational and commercial areas of Chesapeake Bay and Maryland Coastal Bays. PLoS One 9:e89616. doi: 10.1371/journal.pone.0089616, PMID: 24586914 PMC3934932

[ref78] Singh B. Tyagi A. Billekallu Thammegowda N. K. Ansal M. D. (2018). Prevalence and antimicrobial resistance of vibrios of human health significance in inland saline aquaculture areas. Aquac. Res. 49, 2166–2174. doi: 10.1111/are.13672

[ref79] Smith P. (2012). “7 – Antibiotics in aquaculture: reducing their use and maintaining their efficacy” in *Infectious disease in aquaculture* woodhead publishing series in food science, technology and nutrition. ed. Austin B. (Cambridge, UK: Woodhead Publishing), 161–189.

[ref81] Sperling L. Alter T. Huehn S. (2015). Prevalence and antimicrobial resistance of *Vibrio* spp. in retail and farm shrimps in Ecuador. J. Food Prot. 78, 2089–2092. doi: 10.4315/0362-028X.JFP-15-160, PMID: 26555534

[ref82] Stratev D. Fasulkova R. Krumova-Valcheva G. (2023). Incidence, virulence genes and antimicrobial resistance of *Vibrio parahaemolyticus* isolated from seafood. Microb. Pathog. 177:106050. doi: 10.1016/j.micpath.2023.106050, PMID: 36842516

[ref83] Suhartono S. Savin M. Gbur E. E. (2016). Genetic redundancy and persistence of plasmid-mediated trimethoprim/sulfamethoxazole resistant effluent and stream water *Escherichia coli*. Water Res. 103, 197–204. doi: 10.1016/j.watres.2016.07.035, PMID: 27455416

[ref84] Tate H. Ayers S. Nyirabahizi E. Li C. Borenstein S. Young S.. (2022). Prevalence of antimicrobial resistance in select bacteria from retail seafood—United States, 2019. Front. Microbiol. 13:928509. doi: 10.3389/fmicb.2022.928509, PMID: 35814688 PMC9262255

[ref85] Thompson J. R. Randa M. A. Marcelino L. A. Tomita-Mitchell A. Lim E. Polz M. F. (2004). Diversity and dynamics of a north atlantic coastal vibrio community. Appl. Environ. Microbiol. 70, 4103–4110. doi: 10.1128/AEM.70.7.4103-4110.2004, PMID: 15240289 PMC444776

[ref86] Thomsen S. T. Assunção R. Afonso C. Boué G. Cardoso C. Cubadda F.. (2022). Human health risk–benefit assessment of fish and other seafood: a scoping review. Crit. Rev. Food Sci. Nutr. 62, 7479–7502. doi: 10.1080/10408398.2021.1915240, PMID: 33951954

[ref87] Thornber K. Verner-Jeffreys D. Hinchliffe S. Rahman M. M. Bass D. Tyler C. R. (2020). Evaluating antimicrobial resistance in the global shrimp industry. Rev. Aquac. 12, 966–986. doi: 10.1111/raq.12367, PMID: 32612676 PMC7319481

[ref88] U.S. Department of Agriculture and U.S. Department of Health and Human Services (2020). Dietary Guidelines for Americans, 2020-2025 Available at: https://dietaryguidelines.gov/

[ref89] U.S. Food and Drug Administration (FDA) (2019). The National Antimicrobial Resistance Monitoring System: NARMS Integrated Report, 2016–2017. Laurel, MD U.S. Food and Drug Administration

[ref90] Uma A. Rebecca G. (2018). Antibiotic resistance in bacterial isolates from commercial probiotics used in aquaculture. Int. J. Curr. Microbiol. App. Sci. 7, 1737–1743. doi: 10.20546/ijcmas.2018.701.210

[ref91] Wang F. Jiang L. Yang Q. Han F. Chen S. Pu S.. (2011). Prevalence and antimicrobial susceptibility of major foodborne pathogens in imported seafood. J. Food Prot. 74, 1451–1461. doi: 10.4315/0362-028X.JFP-11-146, PMID: 21902913

[ref93] WHO Critically Important Antimicrobials for Human Medicine 6th (2019). Available at: https://www.who.int/publications/i/item/9789241515528 (Accessed October 12, 2022).

[ref94] Zanetti S. Spanu T. Deriu A. Romano L. Sechi L. A. Fadda G. (2001). In vitro susceptibility of *Vibrio* spp. isolated from the environment. Int. J. Antimicrob. Agents 17, 407–409. doi: 10.1016/S0924-8579(01)00307-7, PMID: 11337229

[ref95] Zankari E. Allesøe R. Joensen K. G. Cavaco L. M. Lund O. Aarestrup F. M. (2017). PointFinder: a novel web tool for WGS-based detection of antimicrobial resistance associated with chromosomal point mutations in bacterial pathogens. J. Antimicrob. Chemother. 72, 2764–2768. doi: 10.1093/jac/dkx217, PMID: 29091202 PMC5890747

[ref96] Zhang M. Chen L. Ye C. Yu X. (2018). Co-selection of antibiotic resistance via copper shock loading on bacteria from a drinking water bio-filter. Environ. Pollut. 233, 132–141. doi: 10.1016/j.envpol.2017.09.084, PMID: 29059628

[ref97] Zhang Y. B. Li Y. Sun X. L. (2011). Antibiotic resistance of bacteria isolated from shrimp hatcheries and cultural ponds on Donghai Island, China. Mar. Pollut. Bull. 62, 2299–2307. doi: 10.1016/j.marpolbul.2011.08.048, PMID: 21945557

[ref98] Zhang X.-X. Zhang T. Fang H. H. P. (2009). Antibiotic resistance genes in water environment. Appl. Microbiol. Biotechnol. 82, 397–414. doi: 10.1007/s00253-008-1829-z 19130050

[ref99] Zhang Y. Zheng Z. Chan E. W.-C. Dong N. Xia X. Chen S. (2018). Molecular characterization of qnrVC genes and their novel alleles in *Vibrio* spp. isolated from food products in China. Antimicrob. Agents Chemother. 62, e00529–e00518. doi: 10.1128/AAC.00529-18, PMID: 29661884 PMC6021619

[ref100] Zheng D. Yin G. Liu M. Chen C. Jiang Y. Hou L.. (2021). A systematic review of antibiotics and antibiotic resistance genes in estuarine and coastal environments. Sci. Total Environ. 777:146009. doi: 10.1016/j.scitotenv.2021.146009, PMID: 33676219

