# Supplementary material for: Prevalence and antimicrobial resistance profiles of Vibrio spp. and Enterococcus spp. in retail shrimp in Northern California
Source: Front Microbiol. 2023 Jun 28;14:1192769. doi: 10.3389/fmicb.2023.1192769 (PMC10338826; doi:10.3389/fmicb.2023.1192769)
Supplement: Supplementary file 1 [file Table_1.DOCX]

**Supplementary Table 1.** Summary of antimicrobial susceptibility testing (AST) drug panels and minimum inhibitory concentration (MIC) interpretive criteria.

|  | **Abbreviation** | **Antimicrobial Agent** | **Antimicrobial Class** | **MIC (ug/ml) Interpretive Critera** | | |
| --- | --- | --- | --- | --- | --- | --- |
|  |  |  |  | **Susceptible** | **Intermediate** | **Resistant** |
| *Enterococcus* | STR | Streptomycin | Aminoglycoside | 1000 | - | ≥ 1000 |
|  | KAN | Kanamycin | Aminoglycoside | ≤ 512 | - | ≥ 1024 |
|  | GEN | Gentamicin | Aminoglycoside | 500 | - | ≥ 500 |
|  | VAN | Vancomycin | Glycopeptide | ≤ 4 | 8-16 | ≥ 32 |
|  | LIN | Lincomycin | Lincosamide | ≤ 2 | 4 | ≥ 8 |
|  | DAP | Daptomycin | Lipopeptide | ≤ 4 | - | - |
|  | TYLT | Tylosin tartrate | Macrolide | ≤ 8 | 16 | ≥ 32 |
|  | ERY | Erythromycin | Macrolide | ≤ 0.5 | 1-4 | ≥ 8 |
|  | NIT | Nitrofurantoin | Nitrofuran | ≤ 32 | 64 | ≥ 128 |
|  | LZD | Linezolid | Oxazolidinones | ≤ 2 | 4 | ≥ 8 |
|  | PEN | Penicillin | Penicillin | ≤ 8 | - | ≥ 16 |
|  | CHL | Chloramphenicol | Phenicol | ≤ 8 | 16 | ≥ 32 |
|  | CIP | Ciprofloxacin | Quinolone | ≤ 1 | 2 | ≥ 4 |
|  | SYN | Quinupristin / dalfopristin | Streptogramin | ≤ 1 | 2 | ≥ 4 |
|  | TGC | Tigecycline | Tetracycline | ≤ 4 | 8 | ≥ 16 |
|  | TET | Tetracycline | Tetracycline | ≤ 4 | 8 | ≥ 16 |
| *Vibrio* | GEN | Gentamicin | Aminoglycoside | ≤ 4 | 8 | ≥ 16 |
|  | STR | Streptomycin | Aminoglycoside | - | - | - |
|  | AUG2 | Amoxicilin / clavulanic acid 2:1 ratio | Beta-lactam combination agent | ≤ 8/4 | 16/8 | ≥ 32/16 |
|  | FOX | Cefoxitin | Cephem | ≤ 8 | 16 | ≥ 32 |
|  | AXO | Ceftriaxone | Cephem | - | - | - |
|  | XNL | Ceftiofur | Cephem | - | - | - |
|  | FIS | Sulfisoxazole | Sulfonamide | - | - | - |
|  | SXT | Trimethoprim / sulfamethoxazole | Sulfonamide | ≤ 2/38 | - | ≥ 4/76 |
|  | AZI | Azithromycin | Macrolide | ≤ 2 | - | - |
|  | AMP | Ampicillin | Penicillin | ≤ 8 | 16 | ≥ 32 |
|  | CHL | Chloramphenicol | Phenicol | - | - | - |
|  | CIP | Ciprofloxacin | Quinolone | ≤ 1 | 2 | ≥ 4 |
|  | NAL | Nalidixic Acid | Quinolone | - | - | - |
|  | TET | Tetracycline | Tetracycline | ≤ 4 | 8 | ≥ 16 |

**Supplementary Table 2.** Distribution of *Vibrio* isolates with MICs below the lowest concentration and above the highest concentration for drugs without defined MIC breakpoints.

| Class | Drug | Lowest concentration on MIC panel (ug/mL) | No. isolates with MIC at or below lowest concentration (%) | Highest concentration on MIC panel (ug/mL) | No. isolates with MIC above highest concentration  (%) |
| --- | --- | --- | --- | --- | --- |
| Cephem | Ceftiofur | 0.12 | 55 (50%) | 8 | 7 (6.36%) |
|  | Ceftriaxone | 0.25 | 95 (86.36%) | 64 | 0 (0%) |
| Phenicol | Chloramphenicol | 2 | 105 (95.45%) | 32 | 0 (0%) |
| Quinolone | Nalidixic acid | 0.5 | 85 (77.27%) | 32 | 5 (4.55%) |
| Aminoglycoside | Streptomycin | 2 | 3 (2.73%) | 64 | 4 (3.64%) |
| Folate pathway antagonist | Sulfisoxazole | 16 | 24 (21.81%) | 256 | 24 (21.82%) |
